# The burden of the current curative expenditure of injury in Dalian, China—a study based on the “system of health accounts 2011”

**DOI:** 10.1186/s12889-021-10164-6

**Published:** 2021-01-19

**Authors:** Shu Sun, Liuna Yang, Xinzhu Hu, Yalan Zhu, Boxi Liu, Yunbin Yang, Xin Wang

**Affiliations:** 1 The First Affiliated Hospital of China Medical University, No.155 Nanjing Beijie, Heping District, Shenyang, Liaoning Province P.R. China 110001; 2 School of public health, Songshan Lake National High-tech Industrial Development, Guangdong Medical University, No.1 Xincheng Blvd, Zone, Dongguan, Guangdong Province P.R. China 523808; 3 College of the Humanities and Social Sciences, China Medical University, No.77 Puhe Road, Shenyang North New Area, Shenyang, Liaoning Province P.R. China 110122; 4 Southern Medical University, 1023-1063 Shatai south road, Guangzhou, Guangdong Province P.R. China 510515

**Keywords:** Current curative expenditure, Injury, System of health accounts 2011

## Abstract

**Background:**

Injury is one of the major public health problems and causes more than 5 million deaths in the world annually. Cases of specific types of injury are life-threatening and heavily-burdened to individuals and society. This study was aimed to assess the financial burden of injury on patients.

**Methods:**

A total of 565 medical institutions were selected with multistage stratified cluster random sampling, containing 152,553 valid samples. Subsequently, the distribution of injury current curative expenditure (CCE) in different dimensions (including age and site of injury) was analyzed under the framework of System of Health Accounts 2011 (SHA 2011) using the established database.

**Results:**

In China, both urban and rural injury mortality rates showed an upward trend of more than 5 percentage points from 2006 to 2016. In Dalian, the CCE of injury reached 1572.73 million RMB, accounting for 7.45% of the total CCE. Those aged 15–24 reported larger proportion of CCE than the other age groups. As for the injuries in body parts, injuries occurred to the spine, lower limb, head and foreign body cost most.

**Conclusions:**

Dalian has a relatively serious burden of injury costs. The essential and primary goal is to reduce the cost. Young people should pay attention to protect their head and limbs from injury, and related government sectors should implement preventive and educative measures.

**Supplementary Information:**

The online version contains supplementary material available at 10.1186/s12889-021-10164-6.

## Background

In recent years, the incidence and mortality of injury have shown an increasing trend internationally. Injuries resulting from traffic collisions, drowning, poisoning, falls or burns from assault, self-inflicted violence or acts of war lead to more than five million death worldwide annually and cause harm to millions more [1]. Injury account for 9% of global mortality, and has become a major public health problem to all countries in the world [2]. Injuries are one of the leading causes of death in China, with about 300 million injuries occurring each year, accounting for 11% of mortality [3].

Meanwhile, injury is the leading cause of death among young people. Road traffic injuries are the leading cause of death among people aged 15 to 29, and 1.25 million people died from road traffic injuries in 2013 [4]. Globally, road traffic deaths increased by 13% between 2000 and 2013. It’s estimated that road injuries will lead to 1.8 trillion dollars monetary loss in 2015–2030 worldwide, equivalent to 0.12% annual tax of global gross domestic product (GDP) [5]. Open lower limb fractures are expensive and resource-intensive treatment. The treatment cost 19,200 pounds per patient in England, which is not only great labor force and economic loss, but also stunt to social development [6]. As a result of the high incidence and heavy financial burden of injury, governments around the world have become compelled to find means of decreasing and preventing the high incidence of injuries, due to the significant social impact and financial burden caused by them.

The high CCE of injury in China poses a sizeable challenge. Injuries imposed heavy burdens on individuals and society. According to WHO, the age-standardized Disability Adjusted Life Years (DALYs) per 100,000 population caused by injuries were 31,343 in China [7]. In 2014, medical expenses for unintentional injuries in Sichuan province were 491.15 million RMB [8]. In 2017, the CCE of injury in Gansu province was 3.831 billion RMB [9].

According to *China National Statistical Yearbook 2017*, the mortality of injuries in 2016 for urban residents and rural residents were 37.34 and 54.48 per 100,000 population respectively. Deaths from injuries contributed 6.08% to all deaths for urban residents and 8.01% of all deaths for rural residents [10]. Approximately 50% of deaths in age of 15–30 were caused by injuries [11].

In previous studies, the data of injuries were generally obtained from the Death Registration System of China Center for Disease Control and Prevention or the National Injury Surveillance System (NISS) [12], which were analyzed according to external causes. Most studies’ analyses of the burden of injuries were based on incidence, mortality, cause of death sequence and DALYs. However, those studies lack the information and analysis of treatment and payment. Measuring the cost of injuries and characteristics of distribution is critical to identifying priorities for policies, which is of significant meaning to reducing injuries and their consequences [13–15]. Therefore, further studies on CCE on needed.

System of Health Accounts 2011 (SHA 2011) is a new health care accounts system, which provides a framework to account for the CCE by diseases types and age groups, excluding the expenditure of prevention [16]. In the present study, SHA 2011 was used to analyze the distribution of injury cost in different age groups and in different classifications, in order to assessed the economic burden.

## Methods

### Data source

Macro data were obtained from *Liaoning Health Statistical Yearbook 2017*, *Liaoning Health Financial Annual Reports 2017*, *Dalian Current Health Expenditure Report 2016*, and *China National Health Accounts Report 2017*. The yearbook and annual reports were provided by the Dalian Health Commission. The data of patients’ medical expenses were collected from medical institutions in Dalian by sampling survey. Injury mortality and its percentage of all causes of deaths in China (2006–2016) were extracted from the *China National Statistical Yearbook 2007–2017*.

### Sample data

A total of 565 health institutions were chosen with multistage stratified cluster random sampling from Dalian city of Liaoning Province in 2016. Firstly, nine municipal medical institutions and public health institutions from different districts and counties of Dalian city were selected. Secondly, twenty-one institutions were chosen from each district or county, including one general hospital, one women and children’s hospital, one center for disease control and prevention, one traditional Chinese medicine hospital and 17 clinics. Thirdly, five community health service centers (CHS) and three stations per CHS were selected in each district; twenty township hospitals and three subordinate village clinics were selected in each county. Next, we cleaned up and standardized the key information which was uploaded by these institutions, such as age, gender, disease, International Classification of Disease Tenth Revision (ICD-10) codes, expense, types of insurance, etc. Finally, the state database was created with 4,375,351 valid samples, in which the injury sample was 152,553 after excluding the invalid data. (Additional file 1).

### Classification methods

All samples were divided into eight age groups (≤1, 1–4, 5–14, 15–24, 25–34, 35–44, 45–64, ≥65) [17]. These age groups were divided unevenly, mainly due to the differences in injury profiles (degree and type) among different age brackets [18, 19]. Injuries were classified according to the body parts of injury, based on the codes in chapter 19 of the *International Statistical Classification of Diseases and Related Health Problems, 10th Vision* (ICD-10). ICD code S00-T79 and T90-T97 were selected. Because the number of patients with these two types of injury is very small, we combined “T08-T14 Injuries to unspecified part of trunk, limb or body region” and “T15-T19 Effects of foreign body entering through natural orifice”. In this study, the classification method was used to define different types of injuries, a total of 13 categories including head injury, neck injury and chest injury, etc. (Additional file 2).

### Formula

CCE covers medical income, government basic expenditure subsidy, and government project subsidy income, which is further divided into outpatient and inpatient parts. Taking the expenses of injury as an example (currently no project subsidy directed to this disease, so the calculation is omitted), curative services includes curative income (S_CI_) and basic curative expenditure subsidy (S_BCS_). S_CI_ represents direct medical health expenditure includes treatment fees, medicine fees, diagnosis fees, nursing fees, bed fees, etc. S_BCS_ represents that to ensure the normal operation of the institution and the completion of daily work tasks, the subsidy provided by the finance mainly includes personnel funds and public funds, and the security provision for curative services.

When calculating the CCE, the first step was to exclude the data related to prevention and then ran the following formula to differentiate outpatient and inpatient costs. For detailed procedures, please refer to the previously published literature [16]. All data analysis was performed using STATA12.0. (Additional file 3).

## Results

### Demographic characteristics of participants

A total of 152,553 participants were included in the present study (male vs female = 88,289:64,264; outpatients vs inpatients = 108,615:43,938). Among the participants, 36.89% aged 15–44 and 36.46% aged 45–64. Among the hospitalized patients, 22,218 were hospitalized for 1–3 days, 6245 for 4–7 days, 9383 for 8–15 days, and 6092 for more than 15 days, respectively. Individuals with injury mean expenditures of 3661.268 RMB [95%CI: 3610.479–3712.057].

### Injury mortality and its percentage of all causes of death in China (2006–2016)

Table 1 illustrates the changes of injury-related mortality and composition ratio from 2006 to 2016 in China. In terms of mortality, there increased from 32.36/100,000 in 2006 to 37.34/100,000 in 2017 and from 46.12/100,000 in 2006 to 54.48/100,000 in 2017 for urban and rural residents, respectively. Injuries, as the fifth leading cause of death, contributed a lot to the burden on families and society. Moreover, the mortality and composition ratio of injuries in rural were greater than that in urban (Table 1).

**Table 1:** Injury mortality and its percentage of all causes of death in China (2006–2016)

Year	Urban residents			Rural residents		
	Mortality (per100000)	Percentage(%)	Sequence	Mortality (per100000)	Percentage(%)	Sequence
2006	32.36	6.10	5	46.12	8.90	5
2007	37.63	6.09	5	52.07	8.96	5
2008	31.26	5.08	5	53.02	8.59	5
2009	34.66	5.59	5	54.11	8.25	5
2010	38.09	6.16	5	52.93	8.49	5
2011	33.93	5.47	5	56.50	8.85	5
2012	34.79	5.67	5	58.86	8.92	5
2013	39.01	6.30	5	57.14	8.72	5
2014	37.77	6.13	5	55.29	8.34	5
2015	37.63	6.05	5	53.49	8.07	5
2016	37.34	6.08	5	54.48	8.01	5

### General situation of CCE

In 2016, the CCE for all diseases in Dalian was 21.109 billion RMB, including 7421.92 million RMB (35.16%) for outpatient service and 13,686.90 million RMB (64.84%) for inpatient service. The CCE of injury in Dalian had reached 1572.73 million RMB, accounting for 7.45% of the total curative care expenditure, 0.23% of gross domestic product (GDP).

To identify the distribution of CCE of different age groups, the data were divided into 8 groups and we calculated the CCE both in outpatient and inpatient. The CCE of injuries in outpatient was 356.26 million RMB, accounting for 4.80% of total curative care expenditure in outpatient. The CCE of injury in inpatient was much higher than outpatient, which was 1216.47 million RMB, accounting for 8.89% of total curative care expenditure in inpatient. Overall, injuries were responsible for 7.45% of Dalian’s total burden of disease expenditure.

### The CCE of injuries in different age groups

The CCE of injuries was gradually increasing from newborns to 65 years old, and the age group of 45–64 had the highest CCE of injuries, while the age group less than 1 had the lowest figure. Compared with the other age groups, the CCE of injuries accounted for a larger proportion of total curative care expenditure in the 15–64 age group (Table 2).

**Table 2:** CCE and the CCE of injuries in different age groups (RMB)

Age group	Outpatient			Inpatient		
	CCE (Million)	The CCE of injuries (Million)	Percentage (%)	CCE (Million)	The CCE of injuries (Million)	Percentage (%)
> 1	61.48	0.73	1.19	161.70	2.83	1.75
1–4	365.79	11.26	3.08	299.33	4.26	1.42
5–14	419.65	16.72	3.98	211.15	15.12	7.16
15–24	366.70	25.78	7.03	242.76	62.83	25.88
25–34	1105.36	52.30	4.73	791.67	120.87	15.27
35–44	900.68	57.13	6.34	925.24	160.44	17.34
45–64	2399.59	142.18	5.93	5368.67	504.62	9.40
≥65	1802.66	50.16	2.78	5686.37	345.50	6.08

### CCE for different injuries regions

**Fig. 1:**
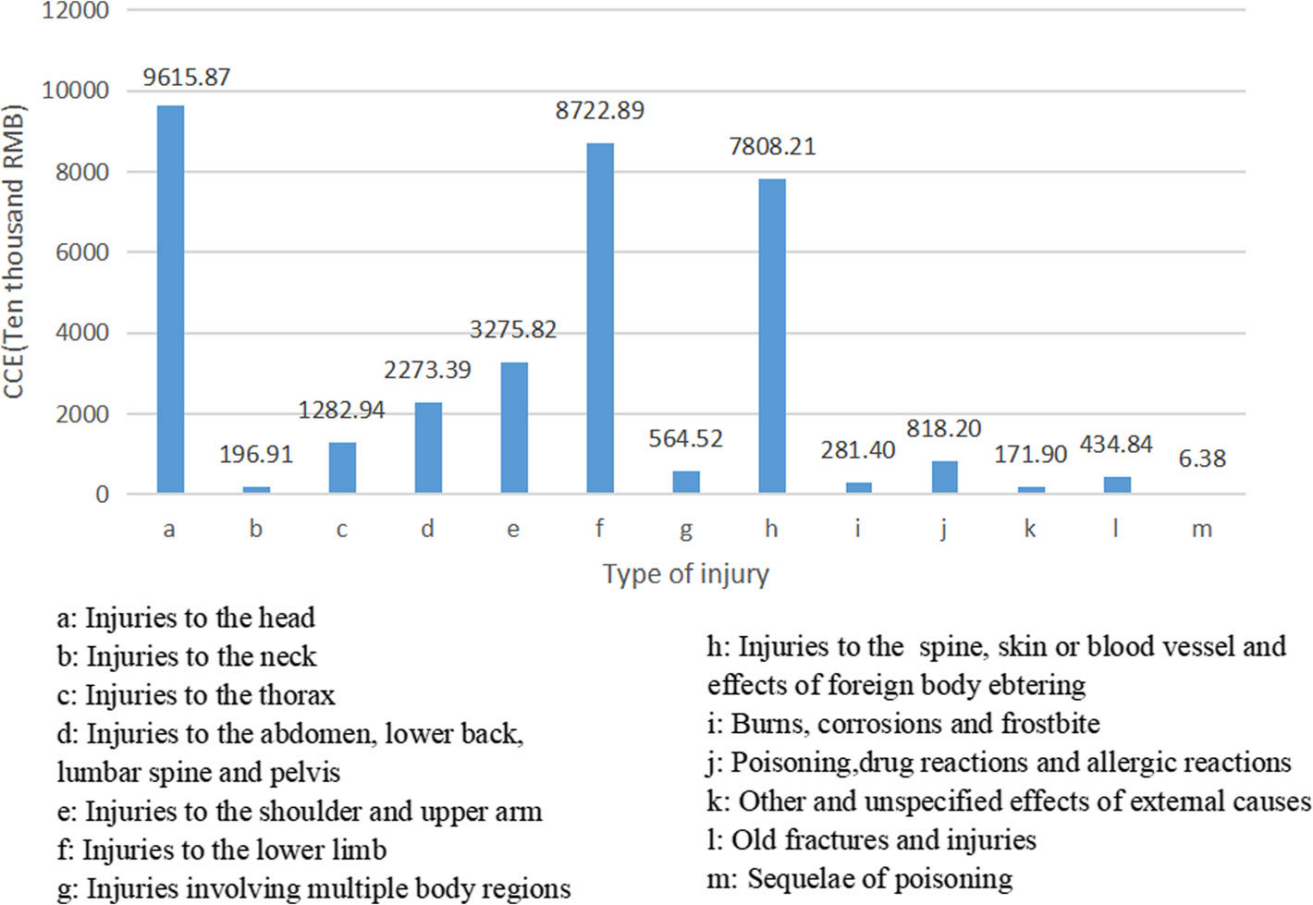
CCE for different injury regions in outpatient. The letters a-m represent 13 types of injuries. The horizontal axis indicates the types of injuries. The longitudinal axis represents the numerical value of CCE

**Fig. 2:**
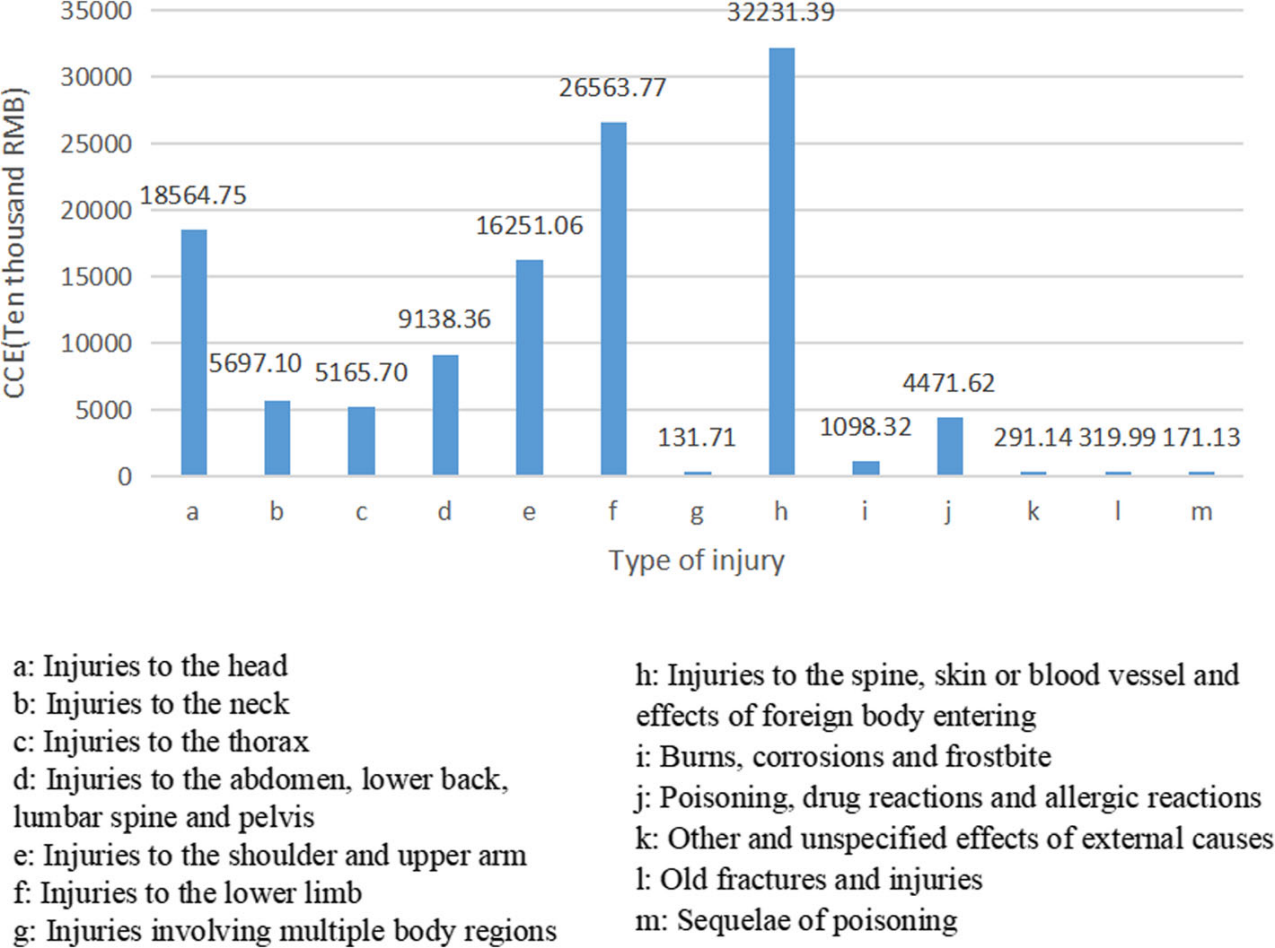
CCE for different injury regions in inpatient. The letters a-m represent 13 types of injuries. The horizontal axis indicates the types of injuries. The longitudinal axis represents the numerical value of CCE

To further understand the distribution of CCE of different types of injuries in the population, we divided injuries into 13 types of according to the injuries on body parts. In terms of injuries of outpatient, the highest expenditure happened in injuries to the head, followed by injuries to the lower limb and injuries to the spine, skin or blood vessel and effects of foreign body insertion. The top three types accounted for 73.39% of the CCE (Fig. 1). However, the top three cost categories of injuries in inpatients care were different, which were injuries to the spine, skin or blood vessel and effects of foreign body insertion, injuries to the lower limb and injuries to the head in inpatient. A total of these three was about 733.60 million RMB, occupying 63.59% of the total in inpatient’s injuries (Fig. 2). The CCE of injuries was 1572.74 million RMB after the outpatient and inpatient were combined, the highest of which was injuries to the spine, skin or blood vessel and effects of foreign body insertion.

### The CCE of different types of injuries for each age group

Table 3 provided a comparison of the CCE of different types of injuries by age groups in outpatient. After analyzing these data, we found that the most costly type of injury in the 0–14 age group was injuries to the spine, skin or blood vessel and effects of foreign body insertion, following by injuries to the head. The most costly type of injury in the 15–64 age group was injuries to the head, following by injuries to the lower limb. In the 65 and above age group, the top two were injuries to the lower limb and injuries to the head (Table 3).

**Table 3:** CCE of different types of injury for each age group in outpatient (Ten thousand RMB)

Type of injury	Age group							
	> 1	1–4	5–14	15–24	25–34	35–44	45–64	≥65
Injuries to the head	23.24	258.62	466.24	784.36	1419.12	1608.11	3857.19	1198.99
Injuries to the neck	1.24	1.11	7.38	7.66	31.28	34.13	74.41	39.69
Injuries to the thorax	1.45	3.63	9.15	54.04	154.67	188.43	618.85	252.72
Injuries to the abdomen, lower back, lumbar spine and pelvis	1.03	4.89	29.00	86.61	263.60	336.97	1059.58	491.70
Injuries to the shoulder and upper arm	2.02	43.81	157.46	236.06	489.88	512.38	1409.77	424.44
Injuries to the lower limb	14.88	139.22	268.20	653.11	1380.95	1511.96	3497.85	1256.73
Injuries involving multiple body regions	0.80	12.23	41.29	21.41	77.55	94.03	245.88	71.33
Injuries to the spine, skin or blood vessel and effects of foreign body insertion	26.46	617.74	616.58	599.13	1195.21	1162.19	2673.99	916.91
Burns, corrosions and frostbite	0.00	15.45	10.34	14.95	24.91	43.40	125.23	47.13
Poisoning, drug reactions and allergic reactions	0.19	15.63	38.57	94.12	132.65	134.23	268.58	134.21
Other and unspecified effects of external causes	1.83	12.57	7.16	14.05	16.85	30.59	50.35	38.51
Old fractures and injuries	0.00	0.17	1.60	4.23	15.52	31.80	271.89	109.63
Sequelae of poisoning	0.00	0.00	1.07	1.76	1.54	2.00	0.00	0.00

As shown in Table 4, the most costly type of injury in the age group 0–14, 15–24 were injuries to the head and injuries to the spine, skin or blood vessel and effects of foreign body insertion, respectively. In addition, the CCE of injuries to the shoulder, upper arm and lower limb should be noticed by the government (Table 4).

**Table 4:** CCE of different types of injury for each age group in inpatient (Ten thousand RMB)

Type of injury	Age group							
	> 1	1–4	5–14	15–24	25–34	35–44	45–64	≥65
Injuries to the head	183.13	217.65	393.17	1077.56	2135.74	2532.87	7989.89	4034.74
Injuries to the neck	8.44	0.00	5.78	49.10	264.79	313.86	2562.80	2492.32
Injuries to the thorax	7.70	9.94	42.85	97.83	245.24	592.08	2419.16	1750.91
Injuries to the abdomen, lower back, lumbar spine and pelvis	0.64	5.91	25.62	456.05	630.90	1309.01	4103.74	2606.48
Injuries to the shoulder and upper arm	0.68	60.75	343.79	1036.39	2287.04	3036.08	6935.01	2551.33
Injuries to the lower limb	25.03	30.71	243.11	1258.90	2266.28	3170.91	11,142.59	8426.26
Injuries involving multiple body regions	0.00	0.00	7.14	3.97	13.49	28.00	59.52	19.59
Injuries to the spine, skin or blood vessel and effects of foreign body insertion	53.37	32.33	288.04	1906.17	3416.66	4081.13	12,054.58	10,399.11
Burns, corrosions and frostbite	2.99	19.89	19.35	77.26	64.41	169.52	575.29	169.62
Poisoning, drug reactions and allergic reactions	1.00	44.90	103.08	228.80	541.51	538.87	1587.84	1425.62
Other and unspecified effects of external causes	0.00	3.89	0.20	2.20	37.89	31.69	95.20	120.07
Old fractures and injuries	0.00	0.00	32.01	8.52	56.83	20.96	128.20	73.45
Sequelae of poisoning	0.00	0.00	0.51	0.00	0.00	5.00	51.63	114.00

## Discussions

To the best of our knowledge, this study was the first to investigate the CCE of injuries in age and site of injury among the Chinese population. This research offered evidences of target population and injuries types that to be the priorities of interventions to reduce the burden of injuries.

### Rising mortality and disparity among urban and rural areas

Injury is the fifth leading cause of death in China and has imposed heavy financial burden to the state. Over the period 2006–2016, mortality of injury has increased with fluctuations in China [20]. When looking at the CCE according to the statistic provided by Dalian City, injuries are major public health issues and the enormous cost burden to sustain. Overall, the mortality and composition ratio of injuries in Chinese rural areas were higher than in urban areas. The finding agreed with the research led by Chunhua He in 2017 indicated that injury mortality among under 5 children in rural areas was higher than in urban areas [21]. It was probably because rural people usually live, work and go to school in an unsafe environment. Meanwhile they benefit less from basic public health services, and have less access to high-quality treatment and rehabilitation services due to the underdeveloped economy.

### CCE in different age groups

The study found that the CCE of injuries in Dalian had reached 1572.73 million RMB, accounting for 7.45% of the total curative care expenditure, 0.23% of GDP [22]. The high expenditure on injury treatment is not only a problem in China, but also in some developed countries. The adjusted national medical cost of injuries was estimated at 56 billion dollars (380.4192 billion RMB) and out-of-pocket cost was approximately 4 billion dollars (27.1728 billion RMB) in the USA in 2016 [23].

In this study, patients aged 45–64 years accounted for 36.46%, so that explains that 41% of CCE injury being spent on 36.46% of injury incidents. This may be due to the large population of this age group, resulting in the high cost of injuries. Based on the data of *China Statistic Yearbook 2017*, the proportion of the population aged 45–64 in the total population has reached to 28.46%, which is the highest in the eight age groups. Another reason may be that middle-aged adults (aged 45–64 years) are more likely to be injured because they undertake more socially productive activities [24]. On the other hand, the CCE of injuries contributed a higher proportion of total curative care expenditure in the 15–44 age group than that in other age groups. The latter situation could be explained by discharge records in 2016 that 36.7% of discharge patients of hospitals in this age group were diagnosed with injuries [10]. This study showed that interventions to reduce CCE should be implemented, targeting people at the age of 15–64.

### Injury burdens among different age groups

For outpatient, the interpretation of the CCE of injuries were divided into three age groups. As for under-15 children, the cost of injuries was mainly caused by injuries to the spine, skin or blood vessel and effects of foreign body insertion and injuries to the head. The top two injuries for people aged 15 to 64 years and over 65 were “head, lower limb” and “lower limb, head” respectively. The probable reason was that people in different age groups were vulnerable to different injuries. Mostly caused by falls, there were high rates of head injury admissions to hospitals occurred among 0–4-year-old (215.5 per 100,000) and people over 65 years of age (188.5 per 100,000) annually [25]. These findings were consistent with the international mainstreams of opinions. For those 70 years or older, falls are the leading category in injury-related deaths. An injury surveillance system pilot study conducted in 4 low/middle-income countries found that falls accounted for the largest percentage (56%) of recorded injuries among children [26]. A study conducted in India similarly found that the most common type of home injury in children aged 0–14 was falling [27]. Children and the elderly were most likely exposed to foreign body and fall-related injuries, in US in 2012, direct medical costs to people over 65 totaled 30.3 billion dollars (approximately 191.268 billion RMB in 2012) for non-fatal injuries in 2012 and rose to 31.3 billion dollars (approximately 195.315 billion RMB in 2015) in 2015 [28]. The inpatient data presented results similar to the outpatient.

### Injuries to the spine, lower limb, head and the effects of foreign bodies

The CCE was different in when injuries happened to different body parts. As results showed, the highest cost happened in injuries to the spine, skin or blood vessel and effects of foreign body insertion, followed by injuries to the lower limb and injuries to the head. It could be attributed to the high frequency and severity of these injury categories [29]. According to National Spinal Cord Injury Statistical Center, the annual incidence of spinal cord injury was approximately 54 cases per one million people in the USA [30], this also indicates that the incidence of spinal injury is higher and is likely to incur more costs, which is similar to the findings of this study. A study carried out in New Zealand showed that head injuries remained a large proportion of injury-related deaths [25], which also supports the conclusion drawn in this study. High treatment costs are expected to be incurred by these patients, especially as the medical costs relating to moribund patients at the end of the period are considerably high. Our results of body regions of injuries are also consistent with those of Zhao Meitao [9]. Research on the injury cost in Gansu Province, China shows that the cost of lower limb injury is as high as 1.09 billion RMB, which is the highest among all injury sites. Lower limb injury has a serious impact on the work and life of residents, and also causes heavy economic burden and social loss. The treatment cost of head injury is 847 million RMB in Gansu in 2017. The special physiological structure of the head leads to serious injuries and consequences after the injury, and the treatment cost is high. Zhao et al. has recommended urgent strengthening of the head safety education and control in traffic and occupational places [9]**.** In terms of injuries to the extremities, extremity fractures had high costs due to high incidences and high productivity loss per patient [31]. Meanwhile, the UK study also shows that open lower limb fractures are expensive to treat at a cost of approximately 16,7961.60 RMB per patient and associate with the severity and area of the limb injury [6].

### Suggestions to decision-makers

Injury has led to high economic costs, and make injury prevention was an enormous challenge in China. Chinese authorities need to allocate more resources to injury prevention. Up to date, National Health Accounts showed government health funding for preventive programs remained minimal [32]. This article calls for increasing funding for injury-related prevention programs. We also highlight the importance of unintentional injury interventions, legislation, and enforcement at a national level. According to the characteristics of injuries in different age groups, the government can propose as a next step to look at the causes of these high-risk age-injury region groups in order to focus prevention strategies, implement targeted interventions in different crowds and then test the effectiveness of them [33]. The study can propose as a next step to look at the causes of these high-risk age and injury part groups before designing preventive strategies. For instance, to protect children from fall-related injuries, schools can install soft rubber and waterproof floor for schoolyards [34].

### Limitation

There were some limitations in this study. Firstly, researchers considered the perfection of health information management system when choosing sample counties, which could have introduced biases. For example, another county-level city in Dalian (Changhai County) was not included in the sample selection area mainly because of its medical conditions were relatively backward, the medical information system was not perfect, so it was unable to obtain complete samples of patients’ treatment information. Secondly, the cost of injuries calculated in this study does not include the cost of subsequent treatment of other diseases resulting from the sequelae of injuries. Because some patients may suffer from a variety of diseases, doctors treat them with a comprehensive consideration of their conditions, so it is difficult to separate out the medical costs associated with complications. In this study, only the patients with the first diagnosis is injuries of all kinds were selected, without considering other complications.

## Conclusion

Injuries were among the most prominent public health problems in the world. The CCE of injuries in Dalian had reached 1572.73 million RMB in 2016, accounting for 7.45% of the total curative care expenditure. People in age group 15–64 and injuries to the spine, lower limb, head and foreign body insertion deserve priorities of interventions.

## Supplementary Information

**Additional file 1.** Sample survey flow chart. The flowchart contains a detailed sampling process.

**Additional file 2.** ICD-10 Appendix to Injury Classification. The appendix contains a detailed code based on the site of the injury.

**Additional file 3.** SHA 2011 Application Appendix. Some of the principles and methods are described in the appendix, which provide a bit more detail about how the SHA 2011 was applied to this work.

## Data Availability

Data was not publically available. The datasets generated during and/or analysed during the current study are available from the corresponding author on reasonable request.

## Abbreviations

WHO: World Health Organization
GDP: Gross domestic product
CCE: Curative care expenditure
DALYs: Disability adjusted life years
NISS: National Injury Surveillance System
SHA 2011: System of Health Accounts 2011
CHS: Community health service center
ICD-10: International Classification of Disease Tenth Revision
