# Supplementary material for: The burden of the current curative expenditure of injury in Dalian, China—a study based on the “system of health accounts 2011”
Source: BMC Public Health. 2021 Jan 19;21:157. doi: 10.1186/s12889-021-10164-6 (PMC7814588; doi:10.1186/s12889-021-10164-6)
Supplement: Supplementary file 1 — Additional file 1. Sample survey flow chart. The flowchart contains a detailed sampling process. [file 12889_2021_10164_MOESM1_ESM.docx]

Sample survey flow chart

5 community health service centres and 15 community health service stations

565 medical institutions

7 districts and 2 county-level cities

35 municipal general hospitals, 29 specialized hospitals and 12 public health institutions

21 institutions in Wa Fangdian

21 institutions in Jinzhou

21 institutions in Xigang

21 institutions in Lv Shunkou

21 institutionsin Pu Landian

21 institutions in Zhuanghe

21 institutions in Gan Jingzi

21 institutions in Sha Hekou

21 instittutions in Zhongshan

20 township health centers

60 village clinics

5 community health service centres and 15 community health service stations

20 township health centers

60 village clinics

5 community health service centres and 15 community health service stations community health service centres and 15 community health service stations5

5 community health service centres and 15 community health service stations

5 community health service centres and 15 community health service stations

5 community health service centres and 15 community health service stations

5 community health service centres and 15 community health service stations

152553 valid samples
