# Supplementary material for: The burden of the current curative expenditure of injury in Dalian, China—a study based on the “system of health accounts 2011”
Source: BMC Public Health. 2021 Jan 19;21:157. doi: 10.1186/s12889-021-10164-6 (PMC7814588; doi:10.1186/s12889-021-10164-6)
Supplement: Supplementary file 2 — Additional file 2. ICD-10 Appendix to Injury Classification. The appendix contains a detailed code based on the site of the injury. [file 12889_2021_10164_MOESM2_ESM.docx]

International Statistical Classification of Diseases and Related Health Problems 10th Revision - Injury

**S00-S09 Injuries to the head**

S00 Superficial injury of head

S00.0 Superficial injury of scalp

S00.1 Contusion of eyelid and periocular area

S00.2 Other superficial injuries of eyelid and periocular area

S00.3 Superficial injury of nose

S00.4 Superficial injury of ear

S00.5 Superficial injury of lip and oral cavity

S00.7 Multiple superficial injuries of head

S00.8 Superficial injury of other parts of head

S00.9 Superficial injury of head, part unspecified

S01 Open wound of head

S01.0 Open wound of scalp

S01.1 Open wound of eyelid and periocular area

S01.2 Open wound of nose

S01.3 Open wound of ear

S01.4 Open wound of cheek and temporomandibular area

S01.5 Open wound of lip and oral cavity

S01.7 Multiple open wounds of head

S01.8 Open wound of other parts of head

S01.9 Open wound of head, part unspecified

S02 Fracture of skull and facial bones

S02.0 Fracture of vault of skull

S02.1 Fracture of base of skull

S02.2 Fracture of nasal bones

S02.3 Fracture of orbital floor

S02.4 Fracture of malar and maxillary bones

S02.5 Fracture of tooth

S02.6 Fracture of mandible

S02.7 Multiple fractures involving skull and facial bones

S02.8 Fractures of other skull and facial bones

S02.9 Fracture of skull and facial bones, part unspecified

S03 Dislocation, sprain and strain of joints and ligaments of head

S03.0 Dislocation of jaw

S03.1 Dislocation of septal cartilage of nose

S03.2 Dislocation of tooth

S03.3 Dislocation of other and unspecified parts of head

S03.4 Sprain and strain of jaw

S03.5 Sprain and strain of joints and ligaments of other and unspecified parts of head

S04 Injury of cranial nerves

S04.0 Injury of optic nerve and pathways

S04.1 Injury of oculomotor nerve

S04.2 Injury of trochlear nerve

S04.3 Injury of trigeminal nerve

S04.4 Injury of abducent nerve

S04.5 Injury of facial nerve

S04.6 Injury of acoustic nerve

S04.7 Injury of accessory nerve

S04.8 Injury of other cranial nerves

S04.9 Injury of unspecified cranial nerve

S05 Injury of eye and orbit

S05.0 Injury of conjunctiva and corneal abrasion without mention of foreign body

S05.1 Contusion of eyeball and orbital tissues

S05.2 Ocular laceration and rupture with prolapse or loss of intraocular tissue

S05.3 Ocular laceration without prolapse or loss of intraocular tissue

S05.4 Penetrating wound of orbit with or without foreign body

S05.5 Penetrating wound of eyeball with foreign body

S05.6 Penetrating wound of eyeball without foreign body

S05.7 Avulsion of eye

S05.8 Other injuries of eye and orbit

S05.9 Injury of eye and orbit, unspecified

S06 Intracranial injury

S06.0 Concussion

S06.1 Traumatic cerebral oedema

S06.2 Diffuse brain injury

S06.3 Focal brain injury

S06.4 Epidural haemorrhage

S06.5 Traumatic subdural haemorrhage

S06.6 Traumatic subarachnoid haemorrhage

S06.7 Intracranial injury with prolonged coma

S06.8 Other intracranial injuries

S06.9 Intracranial injury, unspecified

S07 Crushing injury of head

S07.0 Crushing injury of face

S07.1 Crushing injury of skull

S07.8 Crushing injury of other parts of head

S07.9 Crushing injury of head, part unspecified

S08 Traumatic amputation of part of head

S08.0 Avulsion of scalp

S08.1 Traumatic amputation of ear

S08.8 Traumatic amputation of other parts of head

S08.9 Traumatic amputation of unspecified part of head

S09 Other and unspecified injuries of head

S09.0 Injury of blood vessels of head, not elsewhere classified

S09.1 Injury of muscle and tendon of head

S09.2 Traumatic rupture of ear drum

S09.7 Multiple injuries of head

S09.8 Other specified injuries of head

S09.9 Unspecified injury of head

**S10-S19 Injuries to the neck**

S10 Superficial injury of neck

S10.0 Contusion of throat

S10.1 Other and unspecified superficial injuries of throat

S10.7 Multiple superficial injuries of neck

S10.8 Superficial injury of other parts of neck

S10.9 Superficial injury of neck, part unspecified

S11 Open wound of neck

S11.0 Open wound involving larynx and trachea

S11.1 Open wound involving thyroid gland

S11.2 Open wound involving pharynx and cervical oesophagus

S11.7 Multiple open wounds of neck

S11.8 Open wound of other parts of neck

S11.9 Open wound of neck, part unspecified

S12 Fracture of neck

S12.0 Fracture of first cervical vertebra

S12.1 Fracture of second cervical vertebra

S12.2 Fracture of other specified cervical vertebra

S12.7 Multiple fractures of cervical spine

S12.8 Fracture of other parts of neck

S12.9 Fracture of neck, part unspecified

S13 Dislocation, sprain and strain of joints and ligaments at neck level

S13.0 Traumatic rupture of cervical intervertebral disc

S13.1 Dislocation of cervical vertebra

S13.2 Dislocation of other and unspecified parts of neck

S13.3 Multiple dislocations of neck

S13.4 Sprain and strain of cervical spine

S13.5 Sprain and strain of thyroid region

S13.6 Sprain and strain of joints and ligaments of other and unspecified parts of neck

S14 Injury of nerves and spinal cord at neck level

S14.0 Concussion and oedema of cervical spinal cord

S14.1 Other and unspecified injuries of cervical spinal cord

S14.2 Injury of nerve root of cervical spine

S14.3 Injury of brachial plexus

S14.4 Injury of peripheral nerves of neck

S14.5 Injury of cervical sympathetic nerves

S14.6 Injury of other and unspecified nerves of neck

S15 Injury of blood vessels at neck level

S15.0 Injury of carotid artery

S15.1 Injury of vertebral artery

S15.2 Injury of external jugular vein

S15.3 Injury of internal jugular vein

S15.7 Injury of multiple blood vessels at neck level

S15.8 Injury of other blood vessels at neck level

S15.9 Injury of unspecified blood vessel at neck level

S16 Injury of muscle and tendon at neck level

S17 Crushing injury of neck

S17.0 Crushing injury of larynx and trachea

S17.8 Crushing injury of other parts of neck

S17.9 Crushing injury of neck, part unspecified

S18 Traumatic amputation at neck level

S19 Other and unspecified injuries of neck

S19.7 Multiple injuries of neck

S19.8 Other specified injuries of neck

S19.9 Unspecified injury of neck

**S20-S29 Injuries to the thorax**

S20 Superficial injury of thorax

S20.0 Contusion of breast

S20.1 Other and unspecified superficial injuries of breast

S20.2 Contusion of thorax

S20.3 Other superficial injuries of front wall of thorax

S20.4 Other superficial injuries of back wall of thorax

S20.7 Multiple superficial injuries of thorax

S20.8 Superficial injury of other and unspecified parts of thorax

S21 Open wound of thorax

S21.0 Open wound of breast

S21.1 Open wound of front wall of thorax

S21.2 Open wound of back wall of thorax

S21.7 Multiple open wounds of thoracic wall

S21.8 Open wound of other parts of thorax

S21.9 Open wound of thorax, part unspecified

S22 Fracture of rib(s), sternum and thoracic spine

S22.0 Fracture of thoracic vertebra

S22.1 Multiple fractures of thoracic spine

S22.2 Fracture of sternum

S22.3 Fracture of rib

S22.4 Multiple fractures of ribs

S22.5 Flail chest

S22.8 Fracture of other parts of bony thorax

S22.9 Fracture of bony thorax, part unspecified

S23 Dislocation, sprain and strain of joints and ligaments of thorax

S23.0 Traumatic rupture of thoracic intervertebral disc

S23.1 Dislocation of thoracic vertebra

S23.2 Dislocation of other and unspecified parts of thorax

S23.3 Sprain and strain of thoracic spine

S23.4 Sprain and strain of ribs and sternum

S23.5 Sprain and strain of other and unspecified parts of thorax

S24 Injury of nerves and spinal cord at thorax level

S24.0 Concussion and oedema of thoracic spinal cord

S24.1 Other and unspecified injuries of thoracic spinal cord

S24.2 Injury of nerve root of thoracic spine

S24.3 Injury of peripheral nerves of thorax

S24.4 Injury of thoracic sympathetic nerves

S24.5 Injury of other nerves of thorax

S24.6 Injury of unspecified nerve of thorax

S25 Injury of blood vessels of thorax

S25.0 Injury of thoracic aorta

S25.1 Injury of innominate or subclavian artery

S25.2 Injury of superior vena cava

S25.3 Injury of innominate or subclavian vein

S25.4 Injury of pulmonary blood vessels

S25.5 Injury of intercostal blood vessels

S25.7 Injury of multiple blood vessels of thorax

S25.8 Injury of other blood vessels of thorax

S25.9 Injury of unspecified blood vessel of thorax

S26 Injury of heart

S26.0 Injury of heart with haemopericardium

S26.8 Other injuries of heart

S26.9 Injury of heart, unspecified

S27 Injury of other and unspecified intrathoracic organs

S27.0 Traumatic pneumothorax

S27.1 Traumatic haemothorax

S27.2 Traumatic haemopneumothorax

S27.3 Other injuries of lung

S27.4 Injury of bronchus

S27.5 Injury of thoracic trachea

S27.6 Injury of pleura

S27.7 Multiple injuries of intrathoracic organs

S27.8 Injury of other specified intrathoracic organs

S27.9 Injury of unspecified intrathoracic organ

S28 Crushing injury of thorax and traumatic amputation of part of thorax

S28.0 Crushed chest

S28.1 Traumatic amputation of part of thorax

S29 Other and unspecified injuries of thorax

S29.0 Injury of muscle and tendon at thorax level

S29.7 Multiple injuries of thorax

S29.8 Other specified injuries of thorax

S29.9 Unspecified injury of thorax

**S30-S39 Injuries to the abdomen, lower back, lumbar spine and pelvis**

S30 Superficial injury of abdomen, lower back and pelvis

S30.0 Contusion of lower back and pelvis

S30.1 Contusion of abdominal wall

S30.2 Contusion of external genital organs

S30.7 Multiple superficial injuries of abdomen, lower back and pelvis

S30.8 Other superficial injuries of abdomen, lower back and pelvis

S30.9 Superficial injury of abdomen, lower back and pelvis, part unspecified

S31 Open wound of abdomen, lower back and pelvis

S31.0 Open wound of lower back and pelvis

S31.1 Open wound of abdominal wall

S31.2 Open wound of penis

S31.3 Open wound of scrotum and testes

S31.4 Open wound of vagina and vulva

S31.5 Open wound of other and unspecified external genital organs

S31.7 Multiple open wounds of abdomen, lower back and pelvis

S31.8 Open wound of other and unspecified parts of abdomen

S32 Fracture of lumbar spine and pelvis

S32.0 Fracture of lumbar vertebra

S32.1 Fracture of sacrum

S32.2 Fracture of coccyx

S32.3 Fracture of ilium

S32.4 Fracture of acetabulum

S32.5 Fracture of pubis

S32.7 Multiple fractures of lumbar spine and pelvis

S32.8 Fracture of other and unspecified parts of lumbar spine and pelvis

S33 Dislocation, sprain and strain of joints and ligaments of lumbar spine and pelvis

S33.0 Traumatic rupture of lumbar intervertebral disc

S33.1 Dislocation of lumbar vertebra

S33.2 Dislocation of sacroiliac and sacrococcygeal joint

S33.3 Dislocation of other and unspecified parts of lumbar spine and pelvis

S33.4 Traumatic rupture of symphysis pubis

S33.5 Sprain and strain of lumbar spine

S33.6 Sprain and strain of sacroiliac joint

S33.7 Sprain and strain of other and unspecified parts of lumbar spine and pelvis

S34 Injury of nerves and lumbar spinal cord at abdomen, lower back and pelvis level

S34.0 Concussion and oedema of lumbar spinal cord

S34.1 Other injury of lumbar spinal cord

S34.2 Injury of nerve root of lumbar and sacral spine

S34.3 Injury of cauda equina

S34.4 Injury of lumbosacral plexus

S34.5 Injury of lumbar, sacral and pelvic sympathetic nerves

S34.6 Injury of peripheral nerve(s) of abdomen, lower back and pelvis

S34.8 Injury of other and unspecified nerves at abdomen, lower back and pelvis level

S35 Injury of blood vessels at abdomen, lower back and pelvis level

S35.0 Injury of abdominal aorta

S35.1 Injury of inferior vena cava

S35.2 Injury of coeliac or mesenteric artery

S35.3 Injury of portal or splenic vein

S35.4 Injury of renal blood vessels

S35.5 Injury of iliac blood vessels

S35.7 Injury of multiple blood vessels at abdomen, lower back and pelvis level

S35.8 Injury of other blood vessels at abdomen, lower back and pelvis level

S35.9 Injury of unspecified blood vessel at abdomen, lower back and pelvis level

S36 Injury of intra-abdominal organs

S36.0 Injury of spleen

S36.1 Injury of liver or gallbladder

S36.2 Injury of pancreas

S36.3 Injury of stomach

S36.4 Injury of small intestine

S36.5 Injury of colon

S36.6 Injury of rectum

S36.7 Injury of multiple intra-abdominal organs

S36.8 Injury of other intra-abdominal organs

S36.9 Injury of unspecified intra-abdominal organ

S37 Injury of urinary and pelvic organs

S37.0 Injury of kidney

S37.1 Injury of ureter

S37.2 Injury of bladder

S37.3 Injury of urethra

S37.4 Injury of ovary

S37.5 Injury of fallopian tube

S37.6 Injury of uterus

S37.7 Injury of multiple pelvic organs

S37.8 Injury of other pelvic organs

S37.9 Injury of unspecified pelvic organ

S38 Crushing injury and traumatic amputation of part of abdomen, lower back and pelvis

S38.0 Crushing injury of external genital organs

S38.1 Crushing injury of other and unspecified parts of abdomen, lower back and pelvis

S38.2 Traumatic amputation of external genital organs

S38.3 Traumatic amputation of other and unspecified parts of abdomen, lower back and pelvis

S39 Other and unspecified injuries of abdomen, lower back and pelvis

S39.0 Injury of muscle and tendon of abdomen, lower back and pelvis

S39.6 Injury of intra-abdominal organ(s) with pelvic organ(s)

S39.7 Other multiple injuries of abdomen, lower back and pelvis

S39.8 Other specified injuries of abdomen, lower back and pelvis

S39.9 Unspecified injury of abdomen, lower back and pelvis

**S40-S49 Injuries to the shoulder and upper arm**

S40 Superficial injury of shoulder and upper arm

S40.0 Contusion of shoulder and upper arm

S40.7 Multiple superficial injuries of shoulder and upper arm

S40.8 Other superficial injuries of shoulder and upper arm

S40.9 Superficial injury of shoulder and upper arm, unspecified

S41 Open wound of shoulder and upper arm

S41.0 Open wound of shoulder

S41.1 Open wound of upper arm

S41.7 Multiple open wounds of shoulder and upper arm

S41.8 Open wound of other and unspecified parts of shoulder girdle

S42 Fracture of shoulder and upper arm

S42.0 Fracture of clavicle

S42.1 Fracture of scapula

S42.2 Fracture of upper end of humerus

S42.3 Fracture of shaft of humerus

S42.4 Fracture of lower end of humerus

S42.7 Multiple fractures of clavicle, scapula and humerus

S42.8 Fracture of other parts of shoulder and upper arm

S42.9 Fracture of shoulder girdle, part unspecified

S43 Dislocation, sprain and strain of joints and ligaments of shoulder girdle

S43.0 Dislocation of shoulder joint

S43.1 Dislocation of acromioclavicular joint

S43.2 Dislocation of sternoclavicular joint

S43.3 Dislocation of other and unspecified parts of shoulder girdle

S43.4 Sprain and strain of shoulder joint

S43.5 Sprain and strain of acromioclavicular joint

S43.6 Sprain and strain of sternoclavicular joint

S43.7 Sprain and strain of other and unspecified parts of shoulder girdle

S44 Injury of nerves at shoulder and upper arm level

S44.0 Injury of ulnar nerve at upper arm level

S44.1 Injury of median nerve at upper arm level

S44.2 Injury of radial nerve at upper arm level

S44.3 Injury of axillary nerve

S44.4 Injury of musculocutaneous nerve

S44.5 Injury of cutaneous sensory nerve at shoulder and upper arm level

S44.7 Injury of multiple nerves at shoulder and upper arm level

S44.8 Injury of other nerves at shoulder and upper arm level

S44.9 Injury of unspecified nerve at shoulder and upper arm level

S45 Injury of blood vessels at shoulder and upper arm level

S45.0 Injury of axillary artery

S45.1 Injury of brachial artery

S45.2 Injury of axillary or brachial vein

S45.3 Injury of superficial vein at shoulder and upper arm level

S45.7 Injury of multiple blood vessels at shoulder and upper arm level

S45.8 Injury of other blood vessels at shoulder and upper arm level

S45.9 Injury of unspecified blood vessel at shoulder and upper arm level

S46 Injury of muscle and tendon at shoulder and upper arm level

S46.0 Injury of muscle(s) and tendon(s) of the rotator cuff of shoulder

S46.1 Injury of muscle and tendon of long head of biceps

S46.2 Injury of muscle and tendon of other parts of biceps

S46.3 Injury of muscle and tendon of triceps

S46.7 Injury of multiple muscles and tendons at shoulder and upper arm level

S46.8 Injury of other muscles and tendons at shoulder and upper arm level

S46.9 Injury of unspecified muscle and tendon at shoulder and upper arm level

S47 Crushing injury of shoulder and upper arm

S48 Traumatic amputation of shoulder and upper arm

S48.0 Traumatic amputation at shoulder joint

S48.1 Traumatic amputation at level between shoulder and elbow

S48.9 Traumatic amputation of shoulder and upper arm, level unspecified

S49 Other and unspecified injuries of shoulder and upper arm

S49.7 Multiple injuries of shoulder and upper arm

S49.8 Other specified injuries of shoulder and upper arm

S49.9 Unspecified injury of shoulder and upper arm

**S50-S59 Injuries to the elbow and forearm**

S50 Superficial injury of forearm

S50.0 Contusion of elbow

S50.1 Contusion of other and unspecified parts of forearm

S50.7 Multiple superficial injuries of forearm

S50.8 Other superficial injuries of forearm

S50.9 Superficial injury of forearm, unspecified

S51 Open wound of forearm

S51.0 Open wound of elbow

S51.7 Multiple open wounds of forearm

S51.8 Open wound of other parts of forearm

S51.9 Open wound of forearm, part unspecified

S52 Fracture of forearm

S52.0 Fracture of upper end of ulna

S52.1 Fracture of upper end of radius

S52.2 Fracture of shaft of ulna

S52.3 Fracture of shaft of radius

S52.4 Fracture of shafts of both ulna and radius

S52.5 Fracture of lower end of radius

S52.6 Fracture of lower end of both ulna and radius

S52.7 Multiple fractures of forearm

S52.8 Fracture of other parts of forearm

S52.9 Fracture of forearm, part unspecified

S53 Dislocation, sprain and strain of joints and ligaments of elbow

S53.0 Dislocation of radial head

S53.1 Dislocation of elbow, unspecified

S53.2 Traumatic rupture of radial collateral ligament

S53.3 Traumatic rupture of ulnar collateral ligament

S53.4 Sprain and strain of elbow

S54 Injury of nerves at forearm level

S54.0 Injury of ulnar nerve at forearm level

S54.1 Injury of median nerve at forearm level

S54.2 Injury of radial nerve at forearm level

S54.3 Injury of cutaneous sensory nerve at forearm level

S54.7 Injury of multiple nerves at forearm level

S54.8 Injury of other nerves at forearm level

S54.9 Injury of unspecified nerve at forearm level

S55 Injury of blood vessels at forearm level

S55.0 Injury of ulnar artery at forearm level

S55.1 Injury of radial artery at forearm level

S55.2 Injury of vein at forearm level

S55.7 Injury of multiple blood vessels at forearm level

S55.8 Injury of other blood vessels at forearm level

S55.9 Injury of unspecified blood vessel at forearm level

S56 Injury of muscle and tendon at forearm level

S56.0 Injury of flexor muscle and tendon of thumb at forearm level

S56.1 Injury of long flexor muscle and tendon of other finger(s) at forearm level

S56.2 Injury of other flexor muscle and tendon at forearm level

S56.3 Injury of extensor or abductor muscles and tendons of thumb at forearm level

S56.4 Injury of extensor muscle and tendon of other finger(s) at forearm level

S56.5 Injury of other extensor muscle and tendon at forearm level

S56.7 Injury of multiple muscles and tendons at forearm level

S56.8 Injury of other and unspecified muscles and tendons at forearm level

S57 Crushing injury of forearm

S57.0 Crushing injury of elbow

S57.8 Crushing injury of other parts of forearm

S57.9 Crushing injury of forearm, part unspecified

S58 Traumatic amputation of forearm

S58.0 Traumatic amputation at elbow level

S58.1 Traumatic amputation at level between elbow and wrist

S58.9 Traumatic amputation of forearm, level unspecified

S59 Other and unspecified injuries of forearm

S59.7 Multiple injuries of forearm

S59.8 Other specified injuries of forearm

S59.9 Unspecified injury of forearm

**S60-S69 Injuries to the wrist and hand**

S60 Superficial injury of wrist and hand

S60.0 Contusion of finger(s) without damage to nail

S60.1 Contusion of finger(s) with damage to nail

S60.2 Contusion of other parts of wrist and hand

S60.7 Multiple superficial injuries of wrist and hand

S60.8 Other superficial injuries of wrist and hand

S60.9 Superficial injury of wrist and hand, unspecified

S61 Open wound of wrist and hand

S61.0 Open wound of finger(s) without damage to nail

S61.1 Open wound of finger(s) with damage to nail

S61.7 Multiple open wounds of wrist and hand

S61.8 Open wound of other parts of wrist and hand

S61.9 Open wound of wrist and hand, part unspecified

S62 Fracture at wrist and hand level

S62.0 Fracture of navicular [scaphoid] bone of hand

S62.1 Fracture of other carpal bone(s)

S62.2 Fracture of first metacarpal bone

S62.3 Fracture of other metacarpal bone

S62.4 Multiple fractures of metacarpal bones

S62.5 Fracture of thumb

S62.6 Fracture of other finger

S62.7 Multiple fractures of fingers

S62.8 Fracture of other and unspecified parts of wrist and hand

S63 Dislocation, sprain and strain of joints and ligaments at wrist and hand level

S63.0 Dislocation of wrist

S63.1 Dislocation of finger

S63.2 Multiple dislocations of fingers

S63.3 Traumatic rupture of ligament of wrist and carpus

S63.4 Traumatic rupture of ligament of finger at metacarpophalangeal and interphalangeal joint(s)

S63.5 Sprain and strain of wrist

S63.6 Sprain and strain of finger(s)

S63.7 Sprain and strain of other and unspecified parts of hand

S64 Injury of nerves at wrist and hand level

S64.0 Injury of ulnar nerve at wrist and hand level

S64.1 Injury of median nerve at wrist and hand level

S64.2 Injury of radial nerve at wrist and hand level

S64.3 Injury of digital nerve of thumb

S64.4 Injury of digital nerve of other finger

S64.7 Injury of multiple nerves at wrist and hand level

S64.8 Injury of other nerves at wrist and hand level

S64.9 Injury of unspecified nerve at wrist and hand level

S65 Injury of blood vessels at wrist and hand level

S65.0 Injury of ulnar artery at wrist and hand level

S65.1 Injury of radial artery at wrist and hand level

S65.2 Injury of superficial palmar arch

S65.3 Injury of deep palmar arch

S65.4 Injury of blood vessel(s) of thumb

S65.5 Injury of blood vessel(s) of other finger

S65.7 Injury of multiple blood vessels at wrist and hand level

S65.8 Injury of other blood vessels at wrist and hand level

S65.9 Injury of unspecified blood vessel at wrist and hand level

S66 Injury of muscle and tendon at wrist and hand level

S66.0 Injury of long flexor muscle and tendon of thumb at wrist and hand level

S66.1 Injury of flexor muscle and tendon of other finger at wrist and hand level

S66.2 Injury of extensor muscle and tendon of thumb at wrist and hand level

S66.3 Injury of extensor muscle and tendon of other finger at wrist and hand level

S66.4 Injury of intrinsic muscle and tendon of thumb at wrist and hand level

S66.5 Injury of intrinsic muscle and tendon of other finger at wrist and hand level

S66.6 Injury of multiple flexor muscles and tendons at wrist and hand level

S66.7 Injury of multiple extensor muscles and tendons at wrist and hand level

S66.8 Injury of other muscles and tendons at wrist and hand level

S66.9 Injury of unspecified muscle and tendon at wrist and hand level

S67 Crushing injury of wrist and hand

S67.0 Crushing injury of thumb and other finger(s)

S67.8 Crushing injury of other and unspecified parts of wrist and hand

S68 Traumatic amputation of wrist and hand

S68.0 Traumatic amputation of thumb (complete)(partial)

S68.1 Traumatic amputation of other single finger (complete)(partial)

S68.2 Traumatic amputation of two or more fingers alone (complete)(partial)

S68.3 Combined traumatic amputation of (part of) finger(s) with other parts of wrist and hand

S68.4 Traumatic amputation of hand at wrist level

S68.8 Traumatic amputation of other parts of wrist and hand

S68.9 Traumatic amputation of wrist and hand, level unspecified

S69 Other and unspecified injuries of wrist and hand

S69.7 Multiple injuries of wrist and hand

S69.8 Other specified injuries of wrist and hand

S69.9 Unspecified injury of wrist and hand

**S70-S79 Injuries to the hip and thigh**

S70 Superficial injury of hip and thigh

S70.0 Contusion of hip

S70.1 Contusion of thigh

S70.7 Multiple superficial injuries of hip and thigh

S70.8 Other superficial injuries of hip and thigh

S70.9 Superficial injury of hip and thigh, unspecified

S71 Open wound of hip and thigh

S71.0 Open wound of hip

S71.1 Open wound of thigh

S71.7 Multiple open wounds of hip and thigh

S71.8 Open wound of other and unspecified parts of pelvic girdle

S72 Fracture of femur

S72.0 Fracture of neck of femur

S72.1 Pertrochanteric fracture

S72.2 Subtrochanteric fracture

S72.3 Fracture of shaft of femur

S72.4 Fracture of lower end of femur

S72.7 Multiple fractures of femur

S72.8 Fractures of other parts of femur

S72.9 Fracture of femur, part unspecified

S73 Dislocation, sprain and strain of joint and ligaments of hip

S73.0 Dislocation of hip

S73.1 Sprain and strain of hip

S74 Injury of nerves at hip and thigh level

S74.0 Injury of sciatic nerve at hip and thigh level

S74.1 Injury of femoral nerve at hip and thigh level

S74.2 Injury of cutaneous sensory nerve at hip and thigh level

S74.7 Injury of multiple nerves at hip and thigh level

S74.8 Injury of other nerves at hip and thigh level

S74.9 Injury of unspecified nerve at hip and thigh level

S75 Injury of blood vessels at hip and thigh level

S75.0 Injury of femoral artery

S75.1 Injury of femoral vein at hip and thigh level

S75.2 Injury of greater saphenous vein at hip and thigh level

S75.7 Injury of multiple blood vessels at hip and thigh level

S75.8 Injury of other blood vessels at hip and thigh level

S75.9 Injury of unspecified blood vessel at hip and thigh level

S76 Injury of muscle and tendon at hip and thigh level

S76.0 Injury of muscle and tendon of hip

S76.1 Injury of quadriceps muscle and tendon

S76.2 Injury of adductor muscle and tendon of thigh

S76.3 Injury of muscle and tendon of the posterior muscle group at thigh level

S76.4 Injury of other and unspecified muscles and tendons at thigh level

S76.7 Injury of multiple muscles and tendons at hip and thigh level

S77 Crushing injury of hip and thigh

S77.0 Crushing injury of hip

S77.1 Crushing injury of thigh

S77.2 Crushing injury of hip with thigh

S78 Traumatic amputation of hip and thigh

S78.0 Traumatic amputation at hip joint

S78.1 Traumatic amputation at level between hip and knee

S78.9 Traumatic amputation of hip and thigh, level unspecified

S79 Other and unspecified injuries of hip and thigh

S79.7 Multiple injuries of hip and thigh

S79.8 Other specified injuries of hip and thigh

S79.9 Unspecified injury of hip and thigh

**S80-S89 Injuries to the knee and lower leg**

S80 Superficial injury of lower leg

S80.0 Contusion of knee

S80.1 Contusion of other and unspecified parts of lower leg

S80.7 Multiple superficial injuries of lower leg

S80.8 Other superficial injuries of lower leg

S80.9 Superficial injury of lower leg, unspecified

S81 Open wound of lower leg

S81.0 Open wound of knee

S81.7 Multiple open wounds of lower leg

S81.8 Open wound of other parts of lower leg

S81.9 Open wound of lower leg, part unspecified

S82 Fracture of lower leg, including ankle

S82.0 Fracture of patella

S82.1 Fracture of upper end of tibia

S82.2 Fracture of shaft of tibia

S82.3 Fracture of lower end of tibia

S82.4 Fracture of fibula alone

S82.5 Fracture of medial malleolus

S82.6 Fracture of lateral malleolus

S82.7 Multiple fractures of lower leg

S82.8 Fractures of other parts of lower leg

S82.9 Fracture of lower leg, part unspecified

S83 Dislocation, sprain and strain of joints and ligaments of knee

S83.0 Dislocation of patella

S83.1 Dislocation of knee

S83.2 Tear of meniscus, current

S83.3 Tear of articular cartilage of knee, current

S83.4 Sprain and strain involving (fibular)(tibial) collateral ligament of knee

S83.5 Sprain and strain involving (anterior)(posterior) cruciate ligament of knee

S83.6 Sprain and strain of other and unspecified parts of knee

S83.7 Injury to multiple structures of knee

S84 Injury of nerves at lower leg level

S84.0 Injury of tibial nerve at lower leg level

S84.1 Injury of peroneal nerve at lower leg level

S84.2 Injury of cutaneous sensory nerve at lower leg level

S84.7 Injury of multiple nerves at lower leg level

S84.8 Injury of other nerves at lower leg level

S84.9 Injury of unspecified nerve at lower leg level

S85 Injury of blood vessels at lower leg level

S85.0 Injury of popliteal artery

S85.1 Injury of (anterior)(posterior) tibial artery

S85.2 Injury of peroneal artery

S85.3 Injury of greater saphenous vein at lower leg level

S85.4 Injury of lesser saphenous vein at lower leg level

S85.5 Injury of popliteal vein

S85.7 Injury of multiple blood vessels at lower leg level

S85.8 Injury of other blood vessels at lower leg level

S85.9 Injury of unspecified blood vessel at lower leg level

S86 Injury of muscle and tendon at lower leg level

S86.0 Injury of Achilles tendon

S86.1 Injury of other muscle(s) and tendon(s) of posterior muscle group at lower leg level

S86.2 Injury of muscle(s) and tendon(s) of anterior muscle group at lower leg level

S86.3 Injury of muscle(s) and tendon(s) of peroneal muscle group at lower leg level

S86.7 Injury of multiple muscles and tendons at lower leg level

S86.8 Injury of other muscles and tendons at lower leg level

S86.9 Injury of unspecified muscle and tendon at lower leg level

S87 Crushing injury of lower leg

S87.0 Crushing injury of knee

S87.8 Crushing injury of other and unspecified parts of lower leg

S88 Traumatic amputation of lower leg

S88.0 Traumatic amputation at knee level

S88.1 Traumatic amputation at level between knee and ankle

S88.9 Traumatic amputation of lower leg, level unspecified

S89 Other and unspecified injuries of lower leg

S89.7 Multiple injuries of lower leg

S89.8 Other specified injuries of lower leg

S89.9 Unspecified injury of lower leg

**S90-S99 Injuries to the ankle and foot**

S90 Superficial injury of ankle and foot

S90.0 Contusion of ankle

S90.1 Contusion of toe(s) without damage to nail

S90.2 Contusion of toe(s) with damage to nail

S90.3 Contusion of other and unspecified parts of foot

S90.7 Multiple superficial injuries of ankle and foot

S90.8 Other superficial injuries of ankle and foot

S90.9 Superficial injury of ankle and foot, unspecified

S91 Open wound of ankle and foot

S91.0 Open wound of ankle

S91.1 Open wound of toe(s) without damage to nail

S91.2 Open wound of toe(s) with damage to nail

S91.3 Open wound of other parts of foot

S91.7 Multiple open wounds of ankle and foot

S92 Fracture of foot, except ankle

S92.0 Fracture of calcaneus

S92.1 Fracture of talus

S92.2 Fracture of other tarsal bone(s)

S92.3 Fracture of metatarsal bone

S92.4 Fracture of great toe

S92.5 Fracture of other toe

S92.7 Multiple fractures of foot

S92.9 Fracture of foot, unspecified

S93 Dislocation, sprain and strain of joints and ligaments at ankle and foot level

S93.0 Dislocation of ankle joint

S93.1 Dislocation of toe(s)

S93.2 Rupture of ligaments at ankle and foot level

S93.3 Dislocation of other and unspecified parts of foot

S93.4 Sprain and strain of ankle

S93.5 Sprain and strain of toe(s)

S93.6 Sprain and strain of other and unspecified parts of foot

S94 Injury of nerves at ankle and foot level

S94.0 Injury of lateral plantar nerve

S94.1 Injury of medial plantar nerve

S94.2 Injury of deep peroneal nerve at ankle and foot level

S94.3 Injury of cutaneous sensory nerve at ankle and foot level

S94.7 Injury of multiple nerves at ankle and foot level

S94.8 Injury of other nerves at ankle and foot level

S94.9 Injury of unspecified nerve at ankle and foot level

S95 Injury of blood vessels at ankle and foot level

S95.0 Injury of dorsal artery of foot

S95.1 Injury of plantar artery of foot

S95.2 Injury of dorsal vein of foot

S95.7 Injury of multiple blood vessels at ankle and foot level

S95.8 Injury of other blood vessels at ankle and foot level

S95.9 Injury of unspecified blood vessel at ankle and foot level

S96 Injury of muscle and tendon at ankle and foot level

S96.0 Injury of muscle and tendon of long flexor muscle of toe at ankle and foot level

S96.1 Injury of muscle and tendon of long extensor muscle of toe at ankle and foot level

S96.2 Injury of intrinsic muscle and tendon at ankle and foot level

S96.7 Injury of multiple muscles and tendons at ankle and foot level

S96.8 Injury of other muscles and tendons at ankle and foot level

S96.9 Injury of unspecified muscle and tendon at ankle and foot level

S97 Crushing injury of ankle and foot

S97.0 Crushing injury of ankle

S97.1 Crushing injury of toe(s)

S97.8 Crushing injury of other parts of ankle and foot

S98 Traumatic amputation of ankle and foot

S98.0 Traumatic amputation of foot at ankle level

S98.1 Traumatic amputation of one toe

S98.2 Traumatic amputation of two or more toes

S98.3 Traumatic amputation of other parts of foot

S98.4 Traumatic amputation of foot, level unspecified

S99 Other and unspecified injuries of ankle and foot

S99.7 Multiple injuries of ankle and foot

S99.8 Other specified injuries of ankle and foot

S99.9 Unspecified injury of ankle and foot

**T00-T07 Injuries involving multiple body regions**

T00 Superficial injuries involving multiple body regions

T00.0 Superficial injuries involving head with neck

T00.1 Superficial injuries involving thorax with abdomen, lower back and pelvis

T00.2 Superficial injuries involving multiple regions of upper limb(s)

T00.3 Superficial injuries involving multiple regions of lower limb(s)

T00.6 Superficial injuries involving multiple regions of upper limb(s) with lower limb(s)

T00.8 Superficial injuries involving other combinations of body regions

T00.9 Multiple superficial injuries, unspecified

T01 Open wounds involving multiple body regions

T01.0 Open wounds involving head with neck

T01.1 Open wounds involving thorax with abdomen, lower back and pelvis

T01.2 Open wounds involving multiple regions of upper limb(s)

T01.3 Open wounds involving multiple regions of lower limb(s)

T01.6 Open wounds involving multiple regions of upper limb(s) with lower limb(s)

T01.8 Open wounds involving other combinations of body regions

T01.9 Multiple open wounds, unspecified

T02 Fractures involving multiple body regions

T02.0 Fractures involving head with neck

T02.1 Fractures involving thorax with lower back and pelvis

T02.2 Fractures involving multiple regions of one upper limb

T02.3 Fractures involving multiple regions of one lower limb

T02.4 Fractures involving multiple regions of both upper limbs

T02.5 Fractures involving multiple regions of both lower limbs

T02.6 Fractures involving multiple regions of upper limb(s) with lower limb(s)

T02.7 Fractures involving thorax with lower back and pelvis with limb(s)

T02.8 Fractures involving other combinations of body regions

T02.9 Multiple fractures, unspecified

T03 Dislocations, sprains and strains involving multiple body regions

T03.0 Dislocations, sprains and strains involving head with neck

T03.1 Dislocations, sprains and strains involving thorax with lower back and pelvis

T03.2 Dislocations, sprains and strains involving multiple regions of upper limb(s)

T03.3 Dislocations, sprains and strains involving multiple regions of lower limb(s)

T03.4 Dislocations, sprains and strains involving multiple regions of upper limb(s) with lower limb(s)

T03.8 Dislocations, sprains and strains involving other combinations of body regions

T03.9 Multiple dislocations, sprains and strains, unspecified

T04 Crushing injuries involving multiple body regions

T04.0 Crushing injuries involving head with neck

T04.1 Crushing injuries involving thorax with abdomen, lower back and pelvis

T04.2 Crushing injuries involving multiple regions of upper limb(s)

T04.3 Crushing injuries involving multiple regions of lower limb(s)

T04.4 Crushing injuries involving multiple regions of upper limb(s) with lower limb(s)

T04.7 Crushing injuries of thorax with abdomen, lower back and pelvis with limb(s)

T04.8 Crushing injuries involving other combinations of body regions

T04.9 Multiple crushing injuries, unspecified

T05 Traumatic amputations involving multiple body regions

T05.0 Traumatic amputation of both hands

T05.1 Traumatic amputation of one hand and other arm [any level, except hand]

T05.2 Traumatic amputation of both arms [any level]

T05.3 Traumatic amputation of both feet

T05.4 Traumatic amputation of one foot and other leg [any level, except foot]

T05.5 Traumatic amputation of both legs [any level]

T05.6 Traumatic amputation of upper and lower limbs, any combination [any level]

T05.8 Traumatic amputations involving other combinations of body regions

T05.9 Multiple traumatic amputations, unspecified

T06 Other injuries involving multiple body regions, not elsewhere classified

T06.0 Injuries of brain and cranial nerves with injuries of nerves and spinal cord at neck level

T06.1 Injuries of nerves and spinal cord involving other multiple body regions

T06.2 Injuries of nerves involving multiple body regions

T06.3 Injuries of blood vessels involving multiple body regions

T06.4 Injuries of muscles and tendons involving multiple body regions

T06.5 Injuries of intrathoracic organs with intra-abdominal and pelvic organs

T06.8 Other specified injuries involving multiple body regions

T07 Unspecified multiple injuries

**T08-T14 Injuries to unspecified part of trunk, limb or body region**

T08 Fracture of spine, level unspecified

T09 Other injuries of spine and trunk, level unspecified

T09.0 Superficial injury of trunk, level unspecified

T09.1 Open wound of trunk, level unspecified

T09.2 Dislocation, sprain and strain of unspecified joint and ligament of trunk

T09.3 Injury of spinal cord, level unspecified

T09.4 Injury of unspecified nerve, spinal nerve root and plexus of trunk

T09.5 Injury of unspecified muscle and tendon of trunk

T09.6 Traumatic amputation of trunk, level unspecified

T09.8 Other specified injuries of trunk, level unspecified

T09.9 Unspecified injury of trunk, level unspecified

T10 Fracture of upper limb, level unspecified

T11 Other injuries of upper limb, level unspecified

T11.0 Superficial injury of upper limb, level unspecified

T11.1 Open wound of upper limb, level unspecified

T11.2 Dislocation, sprain and strain of unspecified joint and ligament of upper limb, level unspecified

T11.3 Injury of unspecified nerve of upper limb, level unspecified

T11.4 Injury of unspecified blood vessel of upper limb, level unspecified

T11.5 Injury of unspecified muscle and tendon of upper limb, level unspecified

T11.6 Traumatic amputation of upper limb, level unspecified

T11.8 Other specified injuries of upper limb, level unspecified

T11.9 Unspecified injury of upper limb, level unspecified

T12 Fracture of lower limb, level unspecified

T13 Other injuries of lower limb, level unspecified

T13.0 Superficial injury of lower limb, level unspecified

T13.1 Open wound of lower limb, level unspecified

T13.2 Dislocation, sprain and strain of unspecified joint and ligament of lower limb, level unspecified

T13.3 Injury of unspecified nerve of lower limb, level unspecified

T13.4 Injury of unspecified blood vessel of lower limb, level unspecified

T13.5 Injury of unspecified muscle and tendon of lower limb, level unspecified

T13.6 Traumatic amputation of lower limb, level unspecified

T13.8 Other specified injuries of lower limb, level unspecified

T13.9 Unspecified injury of lower limb, level unspecified

T14 Injury of unspecified body region

T14.0 Superficial injury of unspecified body region

T14.1 Open wound of unspecified body region

T14.2 Fracture of unspecified body region

T14.3 Dislocation, sprain and strain of unspecified body region

T14.4 Injury of nerve(s) of unspecified body region

T14.5 Injury of blood vessel(s) of unspecified body region

T14.6 Injury of muscles and tendons of unspecified body region

T14.7 Crushing injury and traumatic amputation of unspecified body region

T14.8 Other injuries of unspecified body region

T14.9 Injury, unspecified

**T15-T19 Effects of foreign body entering through natural orifice**

T15 Foreign body on external eye

T15.0 Foreign body in cornea

T15.1 Foreign body in conjunctival sac

T15.8 Foreign body in other and multiple parts of external eye

T15.9 Foreign body on external eye, part unspecified

T16 Foreign body in ear

T17 Foreign body in respiratory tract

T17.0 Foreign body in nasal sinus

T17.1 Foreign body in nostril

T17.2 Foreign body in pharynx

T17.3 Foreign body in larynx

T17.4 Foreign body in trachea

T17.5 Foreign body in bronchus

T17.8 Foreign body in other and multiple parts of respiratory tract

T17.9 Foreign body in respiratory tract, part unspecified

T18 Foreign body in alimentary tract

T18.0 Foreign body in mouth

T18.1 Foreign body in oesophagus

T18.2 Foreign body in stomach

T18.3 Foreign body in small intestine

T18.4 Foreign body in colon

T18.5 Foreign body in anus and rectum

T18.8 Foreign body in other and multiple parts of alimentary tract

T18.9 Foreign body in alimentary tract, part unspecified

T19 Foreign body in genitourinary tract

T19.0 Foreign body in urethra

T19.1 Foreign body in bladder

T19.2 Foreign body in vulva and vagina

T19.3 Foreign body in uterus [any part]

T19.8 Foreign body in other and multiple parts of genitourinary tract

T19.9 Foreign body in genitourinary tract, part unspecified

**T20-T32 Burns and corrosions**

T20-T25 Burns and corrosions of external body surface, specified by site

T20 Burn and corrosion of head and neck

T20.0 Burn of unspecified degree of head and neck

T20.1 Burn of first degree of head and neck

T20.2 Burn of second degree of head and neck

T20.3 Burn of third degree of head and neck

T20.4 Corrosion of unspecified degree of head and neck

T20.5 Corrosion of first degree of head and neck

T20.6 Corrosion of second degree of head and neck

T20.7 Corrosion of third degree of head and neck

T21 Burn and corrosion of trunk

T21.0 Burn of unspecified degree of trunk

T21.1 Burn of first degree of trunk

T21.2 Burn of second degree of trunk

T21.3 Burn of third degree of trunk

T21.4 Corrosion of unspecified degree of trunk

T21.5 Corrosion of first degree of trunk

T21.6 Corrosion of second degree of trunk

T21.7 Corrosion of third degree of trunk

T22 Burn and corrosion of shoulder and upper limb, except wrist and hand

T22.0 Burn of unspecified degree of shoulder and upper limb, except wrist and hand

T22.1 Burn of first degree of shoulder and upper limb, except wrist and hand

T22.2 Burn of second degree of shoulder and upper limb, except wrist and hand

T22.3 Burn of third degree of shoulder and upper limb, except wrist and hand

T22.4 Corrosion of unspecified degree of shoulder and upper limb, except wrist and hand

T22.5 Corrosion of first degree of shoulder and upper limb, except wrist and hand

T22.6 Corrosion of second degree of shoulder and upper limb, except wrist and hand

T22.7 Corrosion of third degree of shoulder and upper limb, except wrist and hand

T23 Burn and corrosion of wrist and hand

T23.0 Burn of unspecified degree of wrist and hand

T23.1 Burn of first degree of wrist and hand

T23.2 Burn of second degree of wrist and hand

T23.3 Burn of third degree of wrist and hand

T23.4 Corrosion of unspecified degree of wrist and hand

T23.5 Corrosion of first degree of wrist and hand

T23.6 Corrosion of second degree of wrist and hand

T23.7 Corrosion of third degree of wrist and hand

T24 Burn and corrosion of hip and lower limb, except ankle and foot

T24.0 Burn of unspecified degree of hip and lower limb, except ankle and foot

T24.1 Burn of first degree of hip and lower limb, except ankle and foot

T24.2 Burn of second degree of hip and lower limb, except ankle and foot

T24.3 Burn of third degree of hip and lower limb, except ankle and foot

T24.4 Corrosion of unspecified degree of hip and lower limb, except ankle and foot

T24.5 Corrosion of first degree of hip and lower limb, except ankle and foot

T24.6 Corrosion of second degree of hip and lower limb, except ankle and foot

T24.7 Corrosion of third degree of hip and lower limb, except ankle and foot

T25 Burn and corrosion of ankle and foot

T25.0 Burn of unspecified degree of ankle and foot

T25.1 Burn of first degree of ankle and foot

T25.2 Burn of second degree of ankle and foot

T25.3 Burn of third degree of ankle and foot

T25.4 Corrosion of unspecified degree of ankle and foot

T25.5 Corrosion of first degree of ankle and foot

T25.6 Corrosion of second degree of ankle and foot

T25.7 Corrosion of third degree of ankle and foot

T26-T28 Burns and corrosions confined to eye and internal organs

T26 Burn and corrosion confined to eye and adnexa

T26.0 Burn of eyelid and periocular area

T26.1 Burn of cornea and conjunctival sac

T26.2 Burn with resulting rupture and destruction of eyeball

T26.3 Burn of other parts of eye and adnexa

T26.4 Burn of eye and adnexa, part unspecified

T26.5 Corrosion of eyelid and periocular area

T26.6 Corrosion of cornea and conjunctival sac

T26.7 Corrosion with resulting rupture and destruction of eyeball

T26.8 Corrosion of other parts of eye and adnexa

T26.9 Corrosion of eye and adnexa, part unspecified

T27 Burn and corrosion of respiratory tract

T27.0 Burn of larynx and trachea

T27.1 Burn involving larynx and trachea with lung

T27.2 Burn of other parts of respiratory tract

T27.3 Burn of respiratory tract, part unspecified

T27.4 Corrosion of larynx and trachea

T27.5 Corrosion involving larynx and trachea with lung

T27.6 Corrosion of other parts of respiratory tract

T27.7 Corrosion of respiratory tract, part unspecified

T28 Burn and corrosion of other internal organs

T28.0 Burn of mouth and pharynx

T28.1 Burn of oesophagus

T28.2 Burn of other parts of alimentary tract

T28.3 Burn of internal genitourinary organs

T28.4 Burn of other and unspecified internal organs

T28.5 Corrosion of mouth and pharynx

T28.6 Corrosion of oesophagus

T28.7 Corrosion of other parts of alimentary tract

T28.8 Corrosion of internal genitourinary organs

T28.9 Corrosion of other and unspecified internal organs

T29-T32 Burns and corrosions of multiple and unspecified body regions

T29 Burns and corrosions of multiple body regions

T29.0 Burns of multiple regions, unspecified degree

T29.1 Burns of multiple regions, no more than first-degree burns mentioned

T29.2 Burns of multiple regions, no more than second-degree burns mentioned

T29.3 Burns of multiple regions, at least one burn of third degree mentioned

T29.4 Corrosions of multiple regions, unspecified degree

T29.5 Corrosions of multiple regions, no more than first-degree corrosions mentioned

T29.6 Corrosions of multiple regions, no more than second-degree corrosions mentioned

T29.7 Corrosions of multiple regions, at least one corrosion of third degree mentioned

T30 Burn and corrosion, body region unspecified

T30.0 Burn of unspecified body region, unspecified degree

T30.1 Burn of first degree, body region unspecified

T30.2 Burn of second degree, body region unspecified

T30.3 Burn of third degree, body region unspecified

T30.4 Corrosion of unspecified body region, unspecified degree

T30.5 Corrosion of first degree, body region unspecified

T30.6 Corrosion of second degree, body region unspecified

T30.7 Corrosion of third degree, body region unspecified

T31 Burns classified according to extent of body surface involved

T31.0 Burns involving less than 10% of body surface

T31.1 Burns involving 10-19% of body surface

T31.2 Burns involving 20-29% of body surface

T31.3 Burns involving 30-39% of body surface

T31.4 Burns involving 40-49% of body surface

T31.5 Burns involving 50-59% of body surface

T31.6 Burns involving 60-69% of body surface

T31.7 Burns involving 70-79% of body surface

T31.8 Burns involving 80-89% of body surface

T31.9 Burns involving 90% or more of body surface

T32 Corrosions classified according to extent of body surface involved

T32.0 Corrosions involving less than 10% of body surface

T32.1 Corrosions involving 10-19% of body surface

T32.2 Corrosions involving 20-29% of body surface

T32.3 Corrosions involving 30-39% of body surface

T32.4 Corrosions involving 40-49% of body surface

T32.5 Corrosions involving 50-59% of body surface

T32.6 Corrosions involving 60-69% of body surface

T32.7 Corrosions involving 70-79% of body surface

T32.8 Corrosions involving 80-89% of body surface

T32.9 Corrosions involving 90% or more of body surface

**T33-T35 Frostbite**

T33 Superficial frostbite

T33.0 Superficial frostbite of head

T33.1 Superficial frostbite of neck

T33.2 Superficial frostbite of thorax

T33.3 Superficial frostbite of abdominal wall, lower back and pelvis

T33.4 Superficial frostbite of arm

T33.5 Superficial frostbite of wrist and hand

T33.6 Superficial frostbite of hip and thigh

T33.7 Superficial frostbite of knee and lower leg

T33.8 Superficial frostbite of ankle and foot

T33.9 Superficial frostbite of other and unspecified sites

T34 Frostbite with tissue necrosis

T34.0 Frostbite with tissue necrosis of head

T34.1 Frostbite with tissue necrosis of neck

T34.2 Frostbite with tissue necrosis of thorax

T34.3 Frostbite with tissue necrosis of abdominal wall, lower back and pelvis

T34.4 Frostbite with tissue necrosis of arm

T34.5 Frostbite with tissue necrosis of wrist and hand

T34.6 Frostbite with tissue necrosis of hip and thigh

T34.7 Frostbite with tissue necrosis of knee and lower leg

T34.8 Frostbite with tissue necrosis of ankle and foot

T34.9 Frostbite with tissue necrosis of other and unspecified sites

T35 Frostbite involving multiple body regions and unspecified frostbite

T35.0 Superficial frostbite involving multiple body regions

T35.1 Frostbite with tissue necrosis involving multiple body regions

T35.2 Unspecified frostbite of head and neck

T35.3 Unspecified frostbite of thorax, abdomen, lower back and pelvis

T35.4 Unspecified frostbite of upper limb

T35.5 Unspecified frostbite of lower limb

T35.6 Unspecified frostbite involving multiple body regions

T35.7 Unspecified frostbite of unspecified site

**T36-T50 Poisoning by drugs, medicaments and biological substances**

T36 Poisoning by systemic antibiotics

T36.0 Poisoning: Penicillins

T36.1 Poisoning: Cefalosporins and other beta-lactam antibiotics

T36.2 Poisoning: Chloramphenicol group

T36.3 Poisoning: Macrolides

T36.4 Poisoning: Tetracyclines

T36.5 Poisoning: Aminoglycosides

T36.6 Poisoning: Rifamycins

T36.7 Poisoning: Antifungal antibiotics, systemically used

T36.8 Poisoning: Other systemic antibiotics

T36.9 Poisoning: Systemic antibiotic, unspecified

T37 Poisoning by other systemic anti-infectives and antiparasitics

T37.0 Poisoning: Sulfonamides

T37.1 Poisoning: Antimycobacterial drugs

T37.2 Poisoning: Antimalarials and drugs acting on other blood protozoa

T37.3 Poisoning: Other antiprotozoal drugs

T37.4 Poisoning: Anthelminthics

T37.5 Poisoning: Antiviral drugs

T37.8 Poisoning: Other specified systemic anti-infectives and antiparasitics

T37.9 Poisoning: Systemic anti-infective and antiparasitic, unspecified

T38 Poisoning by hormones and their synthetic substitutes and antagonists, not elsewhere classified

T38.0 Poisoning: Glucocorticoids and synthetic analogues

T38.1 Poisoning: Thyroid hormones and substitutes

T38.2 Poisoning: Antithyroid drugs

T38.3 Poisoning: Insulin and oral hypoglycaemic [antidiabetic] drugs

T38.4 Poisoning: Oral contraceptives

T38.5 Poisoning: Other estrogens and progestogens

T38.6 Poisoning: Antigonadotrophins, antiestrogens, antiandrogens, not elsewhere classified

T38.7 Poisoning: Androgens and anabolic congeners

T38.8 Poisoning: Other and unspecified hormones and their synthetic substitutes

T38.9 Poisoning: Other and unspecified hormone antagonists

T39 Poisoning by nonopioid analgesics, antipyretics and antirheumatics

T39.0 Poisoning: Salicylates

T39.1 Poisoning: 4-Aminophenol derivatives

T39.2 Poisoning: Pyrazolone derivatives

T39.3 Poisoning: Other nonsteroidal anti-inflammatory drugs [NSAID]

T39.4 Poisoning: Antirheumatics, not elsewhere classified

T39.8 Poisoning: Other nonopioid analgesics and antipyretics, not elsewhere classified

T39.9 Poisoning: Nonopioid analgesic, antipyretic and antirheumatic, unspecified

T40 Poisoning by narcotics and psychodysleptics [hallucinogens]

T40.0 Poisoning: Opium

T40.1 Poisoning: Heroin

T40.2 Poisoning: Other opioids

T40.3 Poisoning: Methadone

T40.4 Poisoning: Other synthetic narcotics

T40.5 Poisoning: Cocaine

T40.6 Poisoning: Other and unspecified narcotics

T40.7 Poisoning: Cannabis (derivatives)

T40.8 Poisoning: Lysergide [LSD]

T40.9 Poisoning: Other and unspecified psychodysleptics [hallucinogens]

T41 Poisoning by anaesthetics and therapeutic gases

T41.0 Poisoning: Inhaled anaesthetics

T41.1 Poisoning: Intravenous anaesthetics

T41.2 Poisoning: Other and unspecified general anaesthetics

T41.3 Poisoning: Local anaesthetics

T41.4 Poisoning: Anaesthetic, unspecified

T41.5 Poisoning: Therapeutic gases

T42 Poisoning by antiepileptic, sedative-hypnotic and antiparkinsonism drugs

T42.0 Poisoning: Hydantoin derivatives

T42.1 Poisoning: Iminostilbenes

T42.2 Poisoning: Succinimides and oxazolidinediones

T42.3 Poisoning: Barbiturates

T42.4 Poisoning: Benzodiazepines

T42.5 Poisoning: Mixed antiepileptics, not elsewhere classified

T42.6 Poisoning: Other antiepileptic and sedative-hypnotic drugs

T42.7 Poisoning: Antiepileptic and sedative-hypnotic drugs, unspecified

T42.8 Poisoning: Antiparkinsonism drugs and other central muscle-tone depressants

T43 Poisoning by psychotropic drugs, not elsewhere classified

T43.0 Poisoning: Tricyclic and tetracyclic antidepressants

T43.1 Poisoning: Monoamine-oxidase-inhibitor antidepressants

T43.2 Poisoning: Other and unspecified antidepressants

T43.3 Poisoning: Phenothiazine antipsychotics and neuroleptics

T43.4 Poisoning: Butyrophenone and thioxanthene neuroleptics

T43.5 Poisoning: Other and unspecified antipsychotics and neuroleptics

T43.6 Poisoning: Psychostimulants with abuse potential

T43.8 Poisoning: Other psychotropic drugs, not elsewhere classified

T43.9 Poisoning: Psychotropic drug, unspecified

T44 Poisoning by drugs primarily affecting the autonomic nervous system

T44.0 Poisoning: Anticholinesterase agents

T44.1 Poisoning: Other parasympathomimetics [cholinergics]

T44.2 Poisoning: Ganglionic blocking drugs, not elsewhere classified

T44.3 Poisoning: Other parasympatholytics [anticholinergics and antimuscarinics] and spasmolytics, not elsewhere classified

T44.4 Poisoning: Predominantly alpha-adrenoreceptor agonists, not elsewhere classified

T44.5 Poisoning: Predominantly beta-adrenoreceptor agonists, not elsewhere classified

T44.6 Poisoning: Alpha-adrenoreceptor antagonists, not elsewhere classified

T44.7 Poisoning: Beta-adrenoreceptor antagonists, not elsewhere classified

T44.8 Poisoning: Centrally acting and adrenergic-neuron-blocking agents, not elsewhere classified

T44.9 Poisoning: Other and unspecified drugs primarily affecting the autonomic nervous system

T45 Poisoning by primarily systemic and haematological agents, not elsewhere classified

T45.0 Poisoning: Antiallergic and antiemetic drugs

T45.1 Poisoning: Antineoplastic and immunosuppressive drugs

T45.2 Poisoning: Vitamins, not elsewhere classified

T45.3 Poisoning: Enzymes, not elsewhere classified

T45.4 Poisoning: Iron and its compounds

T45.5 Poisoning: Anticoagulants

T45.6 Poisoning: Fibrinolysis-affecting drugs

T45.7 Poisoning: Anticoagulant antagonists, vitamin K and other coagulants

T45.8 Poisoning: Other primarily systemic and haematological agents

T45.9 Poisoning: Primarily systemic and haematological agent, unspecified

T46 Poisoning by agents primarily affecting the cardiovascular system

T46.0 Poisoning: Cardiac-stimulant glycosides and drugs of similar action

T46.1 Poisoning: Calcium-channel blockers

T46.2 Poisoning: Other antidysrhythmic drugs, not elsewhere classified

T46.3 Poisoning: Coronary vasodilators, not elsewhere classified

T46.4 Poisoning: Angiotensin-converting-enzyme inhibitors

T46.5 Poisoning: Other antihypertensive drugs, not elsewhere classified

T46.6 Poisoning: Antihyperlipidaemic and antiarteriosclerotic drugs

T46.7 Poisoning: Peripheral vasodilators

T46.8 Poisoning: Antivaricose drugs, including sclerosing agents

T46.9 Poisoning: Other and unspecified agents primarily affecting the cardiovascular system

T47 Poisoning by agents primarily affecting the gastrointestinal system

T47.0 Poisoning: Histamine H 2 -receptor antagonists

T47.1 Poisoning: Other antacids and anti-gastric-secretion drugs

T47.2 Poisoning: Stimulant laxatives

T47.3 Poisoning: Saline and osmotic laxatives

T47.4 Poisoning: Other laxatives

T47.5 Poisoning: Digestants

T47.6 Poisoning: Antidiarrhoeal drugs

T47.7 Poisoning: Emetics

T47.8 Poisoning: Other agents primarily affecting the gastrointestinal system

T47.9 Poisoning: Agent primarily affecting the gastrointestinal system, unspecified

T48 Poisoning by agents primarily acting on smooth and skeletal muscles and the respiratory system

T48.0 Poisoning: Oxytocic drugs

T48.1 Poisoning: Skeletal muscle relaxants [neuromuscular blocking agents]

T48.2 Poisoning: Other and unspecified agents primarily acting on muscles

T48.3 Poisoning: Antitussives

T48.4 Poisoning: Expectorants

T48.5 Poisoning: Anti-common-cold drugs

T48.6 Poisoning: Antiasthmatics, not elsewhere classified

T48.7 Poisoning: Other and unspecified agents primarily acting on the respiratory system

T49 Poisoning by topical agents primarily affecting skin and mucous membrane and by ophthalmological, otorhinolaryngological and dental drugs

T49.0 Poisoning: Local antifungal, anti-infective and anti-inflammatory drugs, not elsewhere classified

T49.1 Poisoning: Antipruritics

T49.2 Poisoning: Local astringents and local detergents

T49.3 Poisoning: Emollients, demulcents and protectants

T49.4 Poisoning: Keratolytics, keratoplastics and other hair treatment drugs and preparations

T49.5 Poisoning: Ophthalmological drugs and preparations

T49.6 Poisoning: Otorhinolaryngological drugs and preparations

T49.7 Poisoning: Dental drugs, topically applied

T49.8 Poisoning: Other topical agents

T49.9 Poisoning: Topical agent, unspecified

T50 Poisoning by diuretics and other and unspecified drugs, medicaments and biological substances

T50.0 Poisoning: Mineralocorticoids and their antagonists

T50.1 Poisoning: Loop [high-ceiling] diuretics

T50.2 Poisoning: Carbonic-anhydrase inhibitors, benzothiadiazides and other diuretics

T50.3 Poisoning: Electrolytic, caloric and water-balance agents

T50.4 Poisoning: Drugs affecting uric acid metabolism

T50.5 Poisoning: Appetite depressants

T50.6 Poisoning: Antidotes and chelating agents, not elsewhere classified

T50.7 Poisoning: Analeptics and opioid receptor antagonists

T50.8 Poisoning: Diagnostic agents

T50.9 Poisoning: Other and unspecified drugs, medicaments and biological substances

**T51-T65 Toxic effects of substances chiefly nonmedicinal as to source**

T51 Toxic effect of alcohol

T51.0 Toxic effect: Ethanol

T51.1 Toxic effect: Methanol

T51.2 Toxic effect: 2-Propanol

T51.3 Toxic effect: Fusel oil

T51.8 Toxic effect: Other alcohols

T51.9 Toxic effect: Alcohol, unspecified

T52 Toxic effect of organic solvents

T52.0 Toxic effect: Petroleum products

T52.1 Toxic effect: Benzene

T52.2 Toxic effect: Homologues of benzene

T52.3 Toxic effect: Glycols

T52.4 Toxic effect: Ketones

T52.8 Toxic effect: Other organic solvents

T52.9 Toxic effect: Organic solvent, unspecified

T53 Toxic effect of halogen derivatives of aliphatic and aromatic hydrocarbons

T53.0 Toxic effect: Carbon tetrachloride

T53.1 Toxic effect: Chloroform

T53.2 Toxic effect: Trichloroethylene

T53.3 Toxic effect: Tetrachloroethylene

T53.4 Toxic effect: Dichloromethane

T53.5 Toxic effect: Chlorofluorocarbons

T53.6 Toxic effect: Other halogen derivatives of aliphatic hydrocarbons

T53.7 Toxic effect: Other halogen derivatives of aromatic hydrocarbons

T53.9 Toxic effect: Halogen derivative of aliphatic and aromatic hydrocarbons, unspecified

T54 Toxic effect of corrosive substances

T54.0 Toxic effect: Phenol and phenol homologues

T54.1 Toxic effect: Other corrosive organic compounds

T54.2 Toxic effect: Corrosive acids and acid-like substances

T54.3 Toxic effect: Corrosive alkalis and alkali-like substances

T54.9 Toxic effect: Corrosive substance, unspecified

T55 Toxic effect of soaps and detergents

T56 Toxic effect of metals

T56.0 Toxic effect: Lead and its compounds

T56.1 Toxic effect: Mercury and its compounds

T56.2 Toxic effect: Chromium and its compounds

T56.3 Toxic effect: Cadmium and its compounds

T56.4 Toxic effect: Copper and its compounds

T56.5 Toxic effect: Zinc and its compounds

T56.6 Toxic effect: Tin and its compounds

T56.7 Toxic effect: Beryllium and its compounds

T56.8 Toxic effect: Other metals

T56.9 Toxic effect: Metal, unspecified

T57 Toxic effect of other inorganic substances

T57.0 Toxic effect: Arsenic and its compounds

T57.1 Toxic effect: Phosphorus and its compounds

T57.2 Toxic effect: Manganese and its compounds

T57.3 Toxic effect: Hydrogen cyanide

T57.8 Toxic effect: Other specified inorganic substances

T57.9 Toxic effect: Inorganic substance, unspecified

T58 Toxic effect of carbon monoxide

T59 Toxic effect of other gases, fumes and vapours

T59.0 Toxic effect: Nitrogen oxides

T59.1 Toxic effect: Sulfur dioxide

T59.2 Toxic effect: Formaldehyde

T59.3 Toxic effect: Lacrimogenic gas

T59.4 Toxic effect: Chlorine gas

T59.5 Toxic effect: Fluorine gas and hydrogen fluoride

T59.6 Toxic effect: Hydrogen sulfide

T59.7 Toxic effect: Carbon dioxide

T59.8 Toxic effect: Other specified gases, fumes and vapours

T59.9 Toxic effect: Gases, fumes and vapours, unspecified

T60 Toxic effect of pesticides

T60.0 Toxic effect: Organophosphate and carbamate insecticides

T60.1 Toxic effect: Halogenated insecticides

T60.2 Toxic effect: Other and unspecified insecticides

T60.3 Toxic effect: Herbicides and fungicides

T60.4 Toxic effect: Rodenticides

T60.8 Toxic effect: Other pesticides

T60.9 Toxic effect: Pesticide, unspecified

T61 Toxic effect of noxious substances eaten as seafood

T61.0 Toxic effect: Ciguatera fish poisoning

T61.1 Toxic effect: Scombroid fish poisoning

T61.2 Toxic effect: Other fish and shellfish poisoning

T61.8 Toxic effect: Toxic effect of other seafoods

T61.9 Toxic effect: Toxic effect of unspecified seafood

T62 Toxic effect of other noxious substances eaten as food

T62.0 Toxic effect: Ingested mushrooms

T62.1 Toxic effect: Ingested berries

T62.2 Toxic effect: Other ingested (parts of) plant(s)

T62.8 Toxic effect: Other specified noxious substances eaten as food

T62.9 Toxic effect: Noxious substance eaten as food, unspecified

T63 Toxic effect of contact with venomous animals

T63.0 Toxic effect: Snake venom

T63.1 Toxic effect: Venom of other reptiles

T63.2 Toxic effect: Venom of scorpion

T63.3 Toxic effect: Venom of spider

T63.4 Toxic effect: Venom of other arthropods

T63.5 Toxic effect: Toxic effect of contact with fish

T63.6 Toxic effect: Toxic effect of contact with other marine animals

T63.8 Toxic effect: Toxic effect of contact with other venomous animals

T63.9 Toxic effect: Toxic effect of contact with unspecified venomous animal

T64 Toxic effect of aflatoxin and other mycotoxin food contaminants

T65 Toxic effect of other and unspecified substances

T65.0 Toxic effect: Cyanides

T65.1 Toxic effect: Strychnine and its salts

T65.2 Toxic effect: Tobacco and nicotine

T65.3 Toxic effect: Nitroderivatives and aminoderivatives of benzene and its homologues

T65.4 Toxic effect: Carbon disulfide

T65.5 Toxic effect: Nitroglycerin and other nitric acids and esters

T65.6 Toxic effect: Paints and dyes, not elsewhere classified

T65.8 Toxic effect: Toxic effect of other specified substances

T65.9 Toxic effect: Toxic effect of unspecified substance

**T66-T78 Other and unspecified effects of external causes**

T66 Unspecified effects of radiation

T67 Effects of heat and light

T67.0 Heatstroke and sunstroke

T67.1 Heat syncope

T67.2 Heat cramp

T67.3 Heat exhaustion, anhydrotic

T67.4 Heat exhaustion due to salt depletion

T67.5 Heat exhaustion, unspecified

T67.6 Heat fatigue, transient

T67.7 Heat oedema

T67.8 Other effects of heat and light

T67.9 Effect of heat and light, unspecified

T68 Hypothermia

T69 Other effects of reduced temperature

T69.0 Immersion hand and foot

T69.1 Chilblains

T69.8 Other specified effects of reduced temperature

T69.9 Effect of reduced temperature, unspecified

T70 Effects of air pressure and water pressure

T70.0 Otitic barotrauma

T70.1 Sinus barotrauma

T70.2 Other and unspecified effects of high altitude

T70.3 Caisson disease [decompression sickness]

T70.4 Effects of high-pressure fluids

T70.8 Other effects of air pressure and water pressure

T70.9 Effect of air pressure and water pressure, unspecified

T71 Asphyxiation

T73 Effects of other deprivation

T73.0 Effects of hunger

T73.1 Effects of thirst

T73.2 Exhaustion due to exposure

T73.3 Exhaustion due to excessive exertion

T73.8 Other effects of deprivation

T73.9 Effect of deprivation, unspecified

T74 Maltreatment syndromes

T74.0 Neglect or abandonment

T74.1 Physical abuse

T74.2 Sexual abuse

T74.3 Psychological abuse

T74.8 Other maltreatment syndromes

T74.9 Maltreatment syndrome, unspecified

T75 Effects of other external causes

T75.0 Effects of lightning

T75.1 Drowning and nonfatal submersion

T75.2 Effects of vibration

T75.3 Motion sickness

T75.4 Effects of electric current

T75.8 Other specified effects of other external causes

T76 Unspecified effects of external causes

T78 Adverse effects, not elsewhere classified

T78.0 Anaphylactic shock due to adverse food reaction

T78.1 Other adverse food reactions, not elsewhere classified

T78.2 Anaphylactic shock, unspecified

T78.3 Angioneurotic oedema

T78.4 Allergy, unspecified

T78.8 Other adverse effects, not elsewhere classified

T78.9 Adverse effect, unspecified

**T79-T79 Certain early complications of trauma**

T79 Certain early complications of trauma, not elsewhere classified

T79.0 Air embolism (traumatic)

T79.1 Fat embolism (traumatic)

T79.2 Traumatic secondary and recurrent haemorrhage

T79.3 Post-traumatic wound infection, not elsewhere classified

T79.4 Traumatic shock

T79.5 Traumatic anuria

T79.6 Traumatic ischaemia of muscle

T79.7 Traumatic subcutaneous emphysema

T79.8 Other early complications of trauma

T79.9 Unspecified early complication of trauma

**T80-T88 Complications of surgical and medical care, not elsewhere classified**

T80 Complications following infusion, transfusion and therapeutic injection

T80.0 Air embolism following infusion, transfusion and therapeutic injection

T80.1 Vascular complications following infusion, transfusion and therapeutic injection

T80.2 Infections following infusion, transfusion and therapeutic injection

T80.3 ABO incompatibility reaction

T80.4 Rh incompatibility reaction

T80.5 Anaphylactic shock due to serum

T80.6 Other serum reactions

T80.8 Other complications following infusion, transfusion and therapeutic injection

T80.9 Unspecified complication following infusion, transfusion and therapeutic injection

T81 Complications of procedures, not elsewhere classified

T81.0 Haemorrhage and haematoma complicating a procedure, not elsewhere classified

T81.1 Shock during or resulting from a procedure, not elsewhere classified

T81.2 Accidental puncture and laceration during a procedure, not elsewhere classified

T81.3 Disruption of operation wound, not elsewhere classified

T81.4 Infection following a procedure, not elsewhere classified

T81.5 Foreign body accidentally left in body cavity or operation wound following a procedure

T81.6 Acute reaction to foreign substance accidentally left during a procedure

T81.7 Vascular complications following a procedure, not elsewhere classified

T81.8 Other complications of procedures, not elsewhere classified

T81.9 Unspecified complication of procedure

T82 Complications of cardiac and vascular prosthetic devices, implants and grafts

T82.0 Mechanical complication of heart valve prosthesis

T82.1 Mechanical complication of cardiac electronic device

T82.2 Mechanical complication of coronary artery bypass and valve grafts

T82.3 Mechanical complication of other vascular grafts

T82.4 Mechanical complication of vascular dialysis catheter

T82.5 Mechanical complication of other cardiac and vascular devices and implants

T82.6 Infection and inflammatory reaction due to cardiac valve prosthesis

T82.7 Infection and inflammatory reaction due to other cardiac and vascular devices, implants and grafts

T82.8 Other specified complications of cardiac and vascular prosthetic devices, implants and grafts

T82.9 Unspecified complication of cardiac and vascular prosthetic device, implant and graft

T83 Complications of genitourinary prosthetic devices, implants and grafts

T83.0 Mechanical complication of urinary (indwelling) catheter

T83.1 Mechanical complication of other urinary devices and implants

T83.2 Mechanical complication of graft of urinary organ

T83.3 Mechanical complication of intrauterine contraceptive device

T83.4 Mechanical complication of other prosthetic devices, implants and grafts in genital tract

T83.5 Infection and inflammatory reaction due to prosthetic device, implant and graft in urinary system

T83.6 Infection and inflammatory reaction due to prosthetic device, implant and graft in genital tract

T83.8 Other complications of genitourinary prosthetic devices, implants and grafts

T83.9 Unspecified complication of genitourinary prosthetic device, implant and graft

T84 Complications of internal orthopaedic prosthetic devices, implants and grafts

T84.0 Mechanical complication of internal joint prosthesis

T84.1 Mechanical complication of internal fixation device of bones of limb

T84.2 Mechanical complication of internal fixation device of other bones

T84.3 Mechanical complication of other bone devices, implants and grafts

T84.4 Mechanical complication of other internal orthopaedic devices, implants and grafts

T84.5 Infection and inflammatory reaction due to internal joint prosthesis

T84.6 Infection and inflammatory reaction due to internal fixation device [any site]

T84.7 Infection and inflammatory reaction due to other internal orthopaedic prosthetic devices, implants and grafts

T84.8 Other complications of internal orthopaedic prosthetic devices, implants and grafts

T84.9 Unspecified complication of internal orthopaedic prosthetic device, implant and graft

T85 Complications of other internal prosthetic devices, implants and grafts

T85.0 Mechanical complication of ventricular intracranial (communicating) shunt

T85.1 Mechanical complication of implanted electronic stimulator of nervous system

T85.2 Mechanical complication of intraocular lens

T85.3 Mechanical complication of other ocular prosthetic devices, implants and grafts

T85.4 Mechanical complication of breast prosthesis and implant

T85.5 Mechanical complication of gastrointestinal prosthetic devices, implants and grafts

T85.6 Mechanical complication of other specified internal prosthetic devices, implants and grafts

T85.7 Infection and inflammatory reaction due to other internal prosthetic devices, implants and grafts

T85.8 Other complications of internal prosthetic devices, implants and grafts, not elsewhere classified

T85.9 Unspecified complication of internal prosthetic device, implant and graft

T86 Failure and rejection of transplanted organs and tissues

T86.0 Bone-marrow transplant rejection

T86.1 Kidney transplant failure and rejection

T86.2 Heart transplant failure and rejection

T86.3 Heart-lung transplant failure and rejection

T86.4 Liver transplant failure and rejection

T86.8 Failure and rejection of other transplanted organs and tissues

T86.9 Failure and rejection of unspecified transplanted organ and tissue

T87 Complications peculiar to reattachment and amputation

T87.0 Complications of reattached (part of) upper extremity

T87.1 Complications of reattached (part of) lower extremity

T87.2 Complications of other reattached body part

T87.3 Neuroma of amputation stump

T87.4 Infection of amputation stump

T87.5 Necrosis of amputation stump

T87.6 Other and unspecified complications of amputation stump

T88 Other complications of surgical and medical care, not elsewhere classified

T88.0 Infection following immunization

T88.1 Other complications following immunization, not elsewhere classified

T88.2 Shock due to anaesthesia

T88.3 Malignant hyperthermia due to anaesthesia

T88.4 Failed or difficult intubation

T88.5 Other complications of anaesthesia

T88.6 Anaphylactic shock due to adverse effect of correct drug or medicament properly administered

T88.7 Unspecified adverse effect of drug or medicament

T88.8 Other specified complications of surgical and medical care, not elsewhere classified

T88.9 Complication of surgical and medical care, unspecified

**T90-T98 Sequelae of injuries, of poisoning and of other consequences of external causes**

T90 Sequelae of injuries of head

T90.0 Sequelae of superficial injury of head

T90.1 Sequelae of open wound of head

T90.2 Sequelae of fracture of skull and facial bones

T90.3 Sequelae of injury of cranial nerves

T90.4 Sequelae of injury of eye and orbit

T90.5 Sequelae of intracranial injury

T90.8 Sequelae of other specified injuries of head

T90.9 Sequelae of unspecified injury of head

T91 Sequelae of injuries of neck and trunk

T91.0 Sequelae of superficial injury and open wound of neck and trunk

T91.1 Sequelae of fracture of spine

T91.2 Sequelae of other fracture of thorax and pelvis

T91.3 Sequelae of injury of spinal cord

T91.4 Sequelae of injury of intrathoracic organs

T91.5 Sequelae of injury of intra-abdominal and pelvic organs

T91.8 Sequelae of other specified injuries of neck and trunk

T91.9 Sequelae of unspecified injury of neck and trunk

T92 Sequelae of injuries of upper limb

T92.0 Sequelae of open wound of upper limb

T92.1 Sequelae of fracture of arm

T92.2 Sequelae of fracture at wrist and hand level

T92.3 Sequelae of dislocation, sprain and strain of upper limb

T92.4 Sequelae of injury of nerve of upper limb

T92.5 Sequelae of injury of muscle and tendon of upper limb

T92.6 Sequelae of crushing injury and traumatic amputation of upper limb

T92.8 Sequelae of other specified injuries of upper limb

T92.9 Sequelae of unspecified injury of upper limb

T93 Sequelae of injuries of lower limb

T93.0 Sequelae of open wound of lower limb

T93.1 Sequelae of fracture of femur

T93.2 Sequelae of other fractures of lower limb

T93.3 Sequelae of dislocation, sprain and strain of lower limb

T93.4 Sequelae of injury of nerve of lower limb

T93.5 Sequelae of injury of muscle and tendon of lower limb

T93.6 Sequelae of crushing injury and traumatic amputation of lower limb

T93.8 Sequelae of other specified injuries of lower limb

T93.9 Sequelae of unspecified injury of lower limb

T94 Sequelae of injuries involving multiple and unspecified body regions

T94.0 Sequelae of injuries involving multiple body regions

T94.1 Sequelae of injuries, not specified by body region

T95 Sequelae of burns, corrosions and frostbite

T95.0 Sequelae of burn, corrosion and frostbite of head and neck

T95.1 Sequelae of burn, corrosion and frostbite of trunk

T95.2 Sequelae of burn, corrosion and frostbite of upper limb

T95.3 Sequelae of burn, corrosion and frostbite of lower limb

T95.4 Sequelae of burn and corrosion classifiable only according to extent of body surface involved

T95.8 Sequelae of other specified burn, corrosion and frostbite

T95.9 Sequelae of unspecified burn, corrosion and frostbite

T96 Sequelae of poisoning by drugs, medicaments and biological substances

T97 Sequelae of toxic effects of substances chiefly nonmedicinal as to source

T98 Sequelae of other and unspecified effects of external causes

T98.0 Sequelae of effects of foreign body entering through natural orifice

T98.1 Sequelae of other and unspecified effects of external causes

T98.2 Sequelae of certain early complications of trauma

T98.3 Sequelae of complications of surgical and medical care, not elsewhere classified

The data comes from the World Health Organization website

https://icd.who.int/browse10/2019/en
