# Supplementary material for: The burden of the current curative expenditure of injury in Dalian, China—a study based on the “system of health accounts 2011”
Source: BMC Public Health. 2021 Jan 19;21:157. doi: 10.1186/s12889-021-10164-6 (PMC7814588; doi:10.1186/s12889-021-10164-6)
Supplement: Supplementary file 3 — Additional file 3. SHA 2011 Application Appendix. Some of the principles and methods are described in the appendix, which provide a bit more detail about how the SHA 2011 was applied to this work. [file 12889_2021_10164_MOESM3_ESM.docx]

**Calculating methods of CCE**

The CCE contained curative income and basic expenditure allowance, including outpatient and inpatient. The formula was shown as follows:

S_CCE_ =
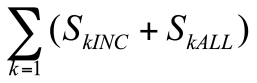
 (1)

In the above formula,
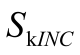
and
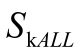
 represented the curative income (Directly expenditure for the patients to see a doctor, including treatment fees, drug fees, examination fees, etc.) and basic expenditure allowance (Government subsidy to the hospital when the patient to see a doctor.) respectively in different medical institutions. The formula of curative income was as follows:

SINC =STINC
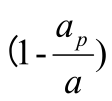
 (2)

SINC
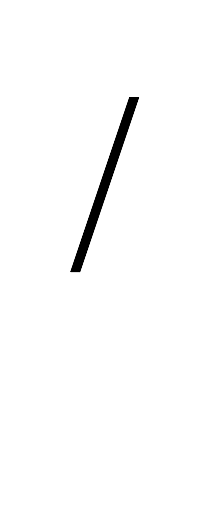
=
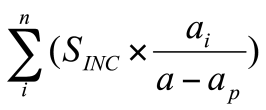
 (3)

Above all the formulas, curative income in per patient from the sample denoted as *a
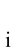
*, total curative income denoted as *a*. Added all sample that contained preventive service based on ICD-10, denoted as *a_p_*, then the sample’s curative income was a
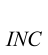
. Per patient sharing coefficient denoted by
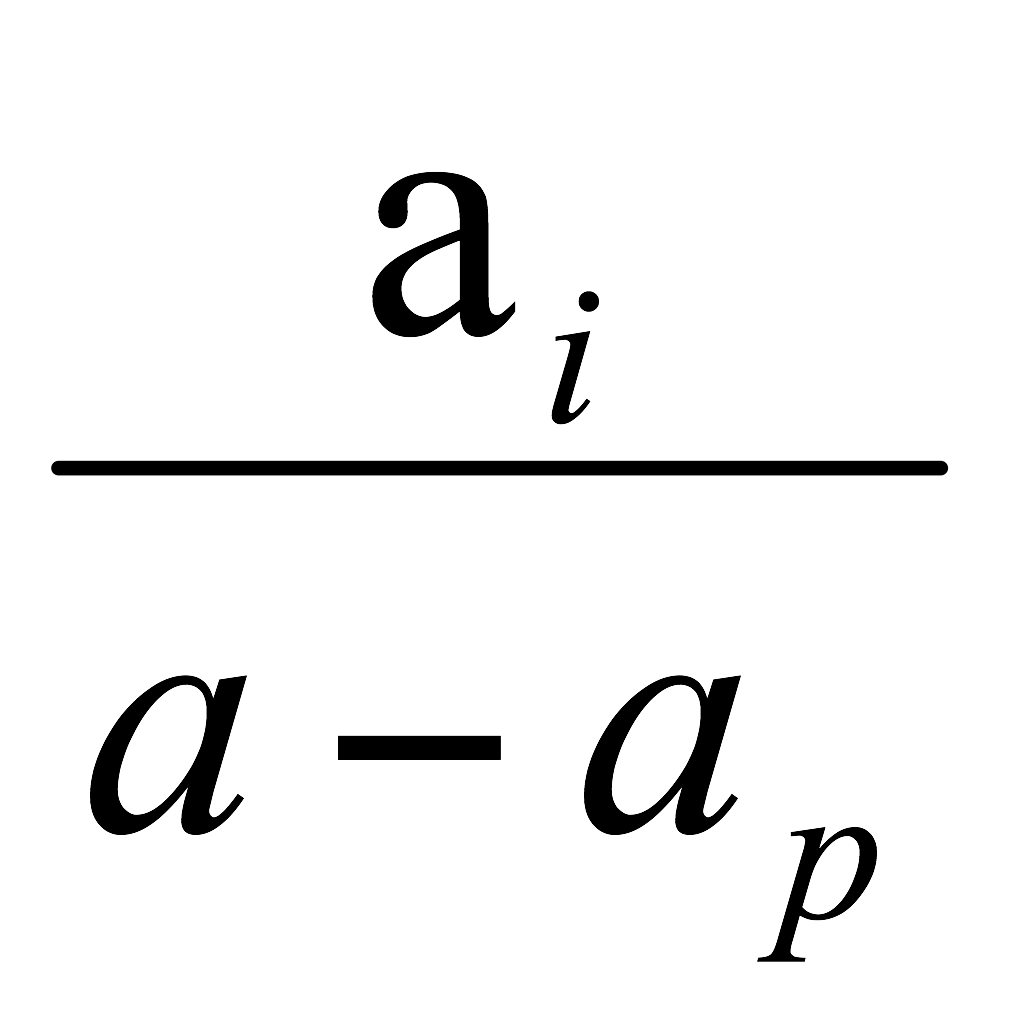
. Moreover, STINC represented the outpatient and inpatient’s income from 2017 Liaoning Health Statistical Yearbook and 2017 Liaoning Health Financial Yearbook. The purpose of formula (2) was to remove prevention expenditure in patient, and the formula of SINC ×
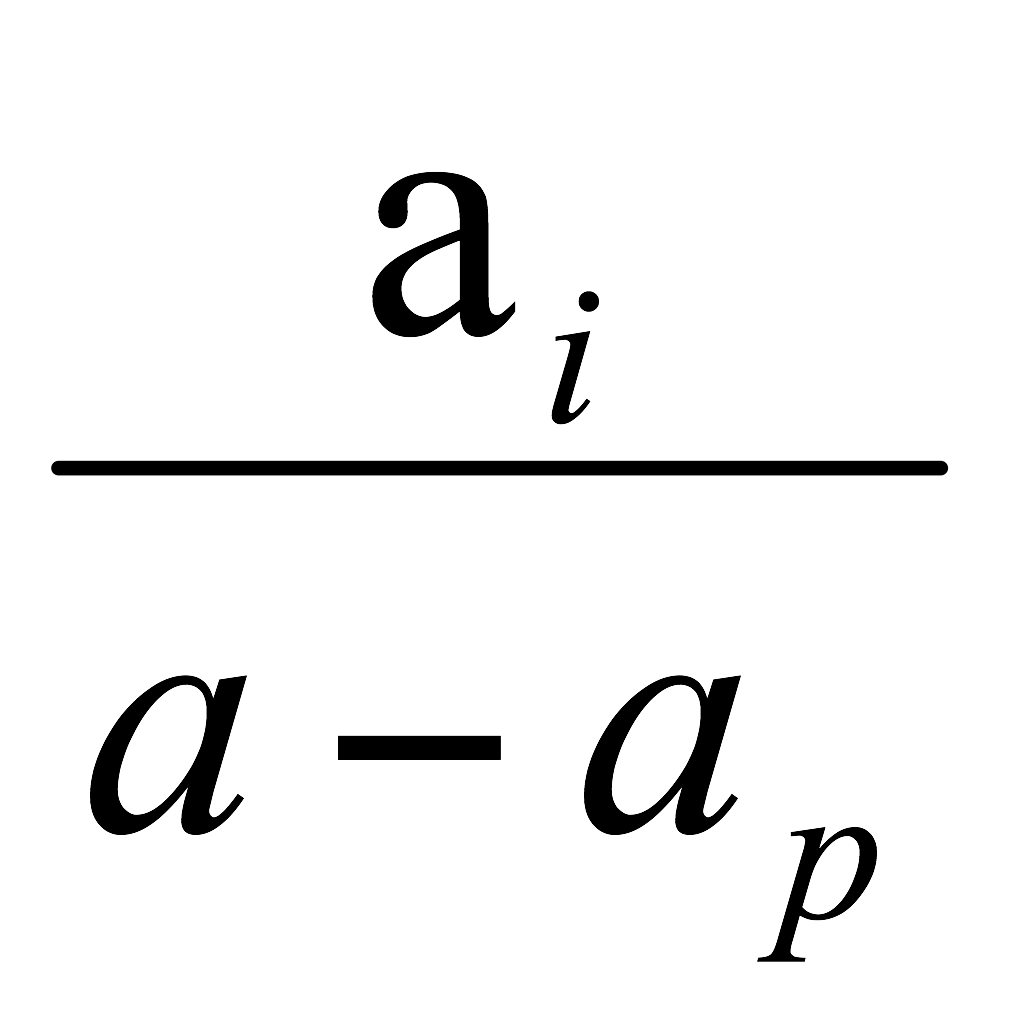
 was to calculate the curative income in patient with the same age, region, etc. The formula (3) indicate that could calculate curative income in various dimensions, such as age, disease, etc. The formula of basic expenditure allowance was as follows;

SALL
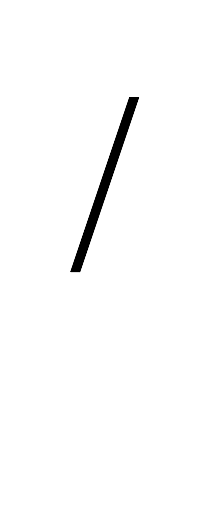
=
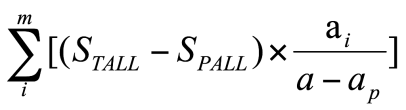
 (4)

Those formulas about basic expenditure allowance were like curative income, the STINC, also collected from basic expenditure allowance from 2017 Liaoning Health Statistical Yearbook. The basic expenditure allowance in preventive service was collected from Yearbook too, denoted as SPALL. STALL − SPALL, meant basic expenditure allowance. The formula (4), split the basic expenditure allowance to per patient. Curative income and basic expenditure allowance of breast cancer patients shared in the above method. Mark off the different dimensions in age, region, etc. The total curative care expenditure in a different age, etc. all could account from the above formula (1).
